# High-resolution transcriptional dissection of *in vivo* Atoh1-mediated hair cell conversion in mature cochleae identifies Isl1 as a co-reprogramming factor

**DOI:** 10.1371/journal.pgen.1007552

**Published:** 2018-07-31

**Authors:** Tetsuji Yamashita, Fei Zheng, David Finkelstein, Zoe Kellard, Robert Carter, Celeste D. Rosencrance, Ken Sugino, John Easton, Charles Gawad, Jian Zuo

**Affiliations:** 1 Department of Developmental Neurobiology, St. Jude Children’s Research Hospital, Memphis, Tennessee, United States of America; 2 Department of Computational Biology, St. Jude Children’s Research Hospital, Memphis, Tennessee, United States of America; 3 Department of Oncology, St. Jude Children’s Research Hospital, Memphis, Tennessee, United States of America; 4 Janelia Farm Research Campus, Howard Hughes Medical Institute, Ashburn, Virginia, United States of America; Stanford University School of Medicine, UNITED STATES

## Abstract

In vivo direct conversion of differentiated cells holds promise for regenerative medicine; however, improving the conversion efficiency and producing functional target cells remain challenging. Ectopic Atoh1 expression in non-sensory supporting cells (SCs) in mouse cochleae induces their partial conversion to hair cells (HCs) at low efficiency. Here, we performed single-cell RNA sequencing of whole mouse sensory epithelia harvested at multiple time points after conditional overexpression of Atoh1. Pseudotemporal ordering revealed that converted HCs (cHCs) are present along a conversion continuum that correlates with both endogenous and exogenous Atoh1 expression. Bulk sequencing of isolated cell populations and single-cell qPCR confirmed 51 transcription factors, including Isl1, are differentially expressed among cHCs, SCs and HCs. In transgenic mice, co-overexpression of Atoh1 and Isl1 enhanced the HC conversion efficiency. Together, our study shows how high-resolution transcriptional profiling of direct cell conversion can identify co-reprogramming factors required for efficient conversion.

## Author summary

The ongoing ATOH1 gene therapy clinical trial offers promise for hearing restoration in humans. However, in animal models, Atoh1-mediated sensory regeneration is inefficient and incomplete. Here we performed high-resolution gene expression profiling of single cochlear cells at multiple time points in a mouse model whereby we discovered a continuous regeneration process that leads to the formation of immature sensory cells. We identified 51 key reprogramming transcription factors that may increase the efficiency and completion of the regeneration process and confirmed that Isl1 in transgenic mice promotes Atoh1-mediated sensory regeneration as a co-reprogramming factor. Our studies identify molecular mechanisms and novel co-reprogramming factors for sensory restoration in humans with irreversible hearing loss.

## Introduction

During development, pluripotent stem cells follow lineage-specific pathways to differentiate into mature cells that can be converted back to pluripotent cells by defined transcription factors (TFs) [1]. In addition, direct lineage conversion (also termed transdifferentiation) between differentiated cells has been demonstrated in heart, pancreas, brain, and other tissues through the use of defined TFs [2–5] or pharmacologic agents [6]. Such conversions have provided a deeper mechanistic understanding of development and hold promise for regenerative medicine. Several studies have used single-cell RNA-seq to identify distinct intermediate conversion states, providing valuable insights into how to improve the efficiency and complete the direct conversions [4, 7, 8].

The cells of the mouse inner-ear cochlear sensory epithelium (organ of Corti) are post-mitotic after birth and exhibit limited spontaneous regeneration that is only present during the first week after birth [9–11]. Atoh1, a lineage-specific TF for sensory hair cells (HCs), directly converts non-sensory supporting cells (SCs) to HCs in cochlear explant culture, as well as in vivo [12–15]. The current model is that ectopic Atoh1 induces the expression of endogenous Atoh1 in SCs to initiate the conversion. This Atoh1-mediated HC conversion is analogous to the natural HC regeneration in chicken inner ears or mammalian vestibular organs [16, 17]. However, the Atoh1-converted HCs (cHCs) in mouse cochleae exhibit immature morphology and do not express several terminal differentiation markers (e.g., *Slc26a5* encoding prestin and *Ocm* encoding oncomodulin). In addition, the process is inefficient, with conversion rates of 6%–20% [13, 14]. Consequently, a more precise understanding of the molecular events underlying Atoh1-induced HC conversion is needed to identify additional factors required for improving the efficiency and completion of the conversion.

In this study, we performed unbiased transcriptional profiling of all cells present in the organ of Corti during Atoh1-mediated SC-to-HC conversion at multiple time points in vivo. This high-resolution transcriptomic analysis revealed new mechanisms of HC conversion in vivo and identified co-reprogramming factors.

## Results

### Single-cell RNA-seq of organs of Corti from juvenile and adult mice during conversion

**Fig 1:**
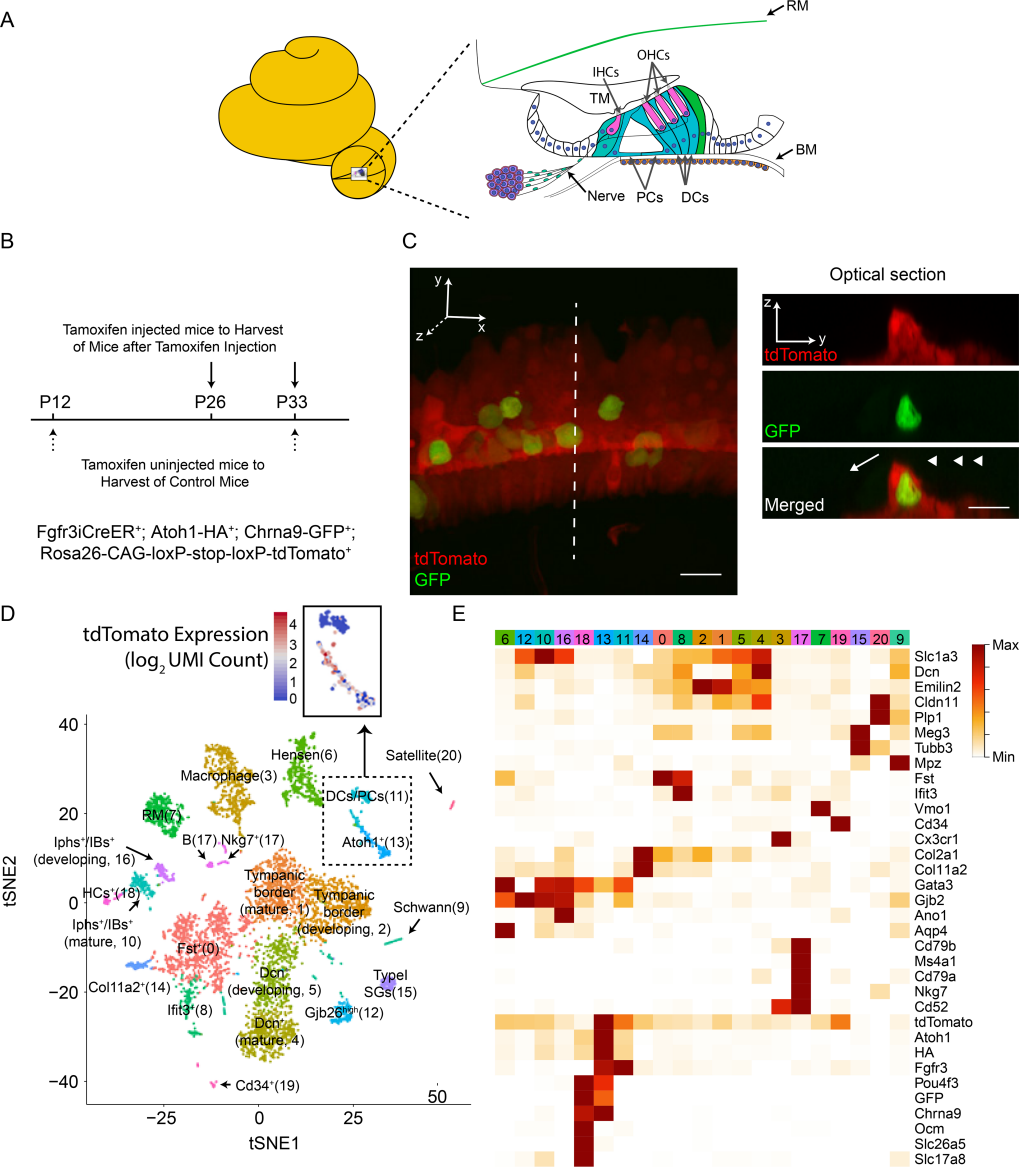
Single-cell gene expression profiling of cochleae during Atoh1-mediated conversion. (A) The area of cochlear cross-section used for single-cell RNA-seq is shown. Note that the lateral walls of cochleae were removed using tweezers. (B) Summary of single-cell RNA-seq experiments. Ages of profiling either with (arrows) or without (dotted arrows) tamoxifen injection are indicated. The mouse used in this assay is indicated. Two biological replicates were used for each group. (C) SCs (PCs/DCs) labeled with tdTomato (red) and cHCs labeled with both tdTomato and EGFP. tdTomato (red) and EGFP (green) expression in Fgfr3iCre^+^; Atoh1-HA^+^; Chrna9-GFP^+^; Rosa26-CAG-loxP-stop-loxP-tdTomato^+^ cochleae from mice at P33 when induced with tamoxifen at P12 [14, 49]. Right panels show a tdTomato^+^/GFP^+^ optical section of a cHC in the dotted line of the cochlear wholemount. Arrowheads indicate positions of three rows of OHCs and the arrow indicates the position of one row of IHCs. Scale bars: 20 μm. (D) PCA followed by tSNE analysis for all 5,470 cochlear cells. Different clusters of cells are identified using distinct colors. RM: Reisner membrane, DCs: Deiters’ cells, PCs: pillar cells, HCs: hair cells, Iphs: inner phalangeal cells, IBs: inner border cells, SGs: spiral ganglia. The tdTomato positive cells are all included in the square with a dashed border and the tdTomato expression level (log_2_ UMI Count) is shown as an inset. (E) Heatmap of selected marker genes in different clusters obtained in **Fig 1D.** Red (maximum value for each gene in log_2_(expected count + 1)) to white (minimum value for each gene in log_2_(expected count + 1)) are shown in means. Rows indicate marker genes while columns indicate different clusters. The colors in columns correspond to those in **Fig 1D**.

In contrast to other regenerative systems, the organ of Corti in the mature cochlea contains relatively few cells: approximately 3,100 HCs [18], including both inner HCs (IHCs) and outer HCs (OHCs), similar numbers of Deiters’ cells (DCs) and pillar cells (PCs) surrounding the OHCs, as well as several other SC subtypes surrounding the IHCs (**Fig 1A**). Massively parallel single-cell RNA sequencing using droplet microfluidics has been shown to be an efficient strategy for acquiring transcriptional profiles from rare cells isolated from fragile structures, as was established in the initial drop-seq study of the human retina [19]. These techniques allow for the rapid and accurate quantification of 5–10% of the transcripts isolated from each cell, which can be expanded upon by identifying and grouping cells with distinct expression programs to create a composite profile of that cell state [20]. We leveraged the technology and these principles to acquire unbiased transcriptional profiles of cells present in the organ of Corti isolated from mouse cochleae.

For HC conversion, we used the previously established mouse model Fgfr3-iCreER; Atoh1-HA; Chrna9-EGFP; tdTomato where ectopic expression of Atoh1-HA transgene was driven by Fgfr3-iCreER-mediated CAG promoter in DCs and PCs after tamoxifen-mediated induction at postnatal day 12 (P12) and P26 and P33 cHCs were identified by their double-positivity for the reporter tdTomato driven by the Fgfr3-iCreER-mediated CAG promoter and EGFP driven by the promotor of endogenous HC gene Chrna9 [14, 21, 22] (**Fig 1A and 1C**). After dissecting and dissociating cells from organs of Corti at P12, P26 and P33, we obtained a total of 5,470 single-cell RNA-seq profiles from 8 organs of Corti that had undergone tamoxifen induction, as well as those from uninduced control mice (**Fig 1B**). We then reduced the dimensionality of the expression matrix and placed individual cells into two-dimensional space using t-Distributed Stochastic Neighbor Embedding (tSNE) (**S1A Fig; Figure Legends**).

Cluster analyses using shared nearest-neighbor modularity optimization-based clustering [23] revealed 21 distinct cell clusters (**Fig 1D; Figure Legends**). Based on expression of known markers for each cochlear cell type, we designated these clusters as HCs (Cluster 18), DCs/PCs (Cluster 11), Hensen cells (Cluster 6), type I spiral ganglia (Cluster 15), tympanic membrane border cells (Cluster 1 for mature cells and Cluster 2 for developing cells), Reissner's membrane cells (Cluster 7), and immune and other unknown cell clusters (**Fig 1D and 1E and S1B Fig and S1 Table**). Focusing on the cells that undergo conversion, we first defined the cluster of DCs/PCs that expressed both endogenous Fgfr3 as well as the Fgfr3-iCreER-mediated reporter (tdTomato) (**Fig 1D** and **S1B Fig**). Near those cells, we identified the cHCs, which expressed both the Atoh1-HA transgene, as well as endogenous Atoh1. The clusters of endogenous OHCs and IHCs were identified based on expression of known markers Ocm and Slc17a8, respectively.

In general, the cells clustered based on cell type and the postnatal age of the mice, suggesting our experimental and analysis pipelines separated cells based on biological differences between the cells (**S1 Fig**). Further supporting the reproducibility of our approach, relative cell cluster frequencies were similar between replicates (**S1C Fig**).

### Identification of a continuum of Atoh1-mediated conversion

**Fig 2:**
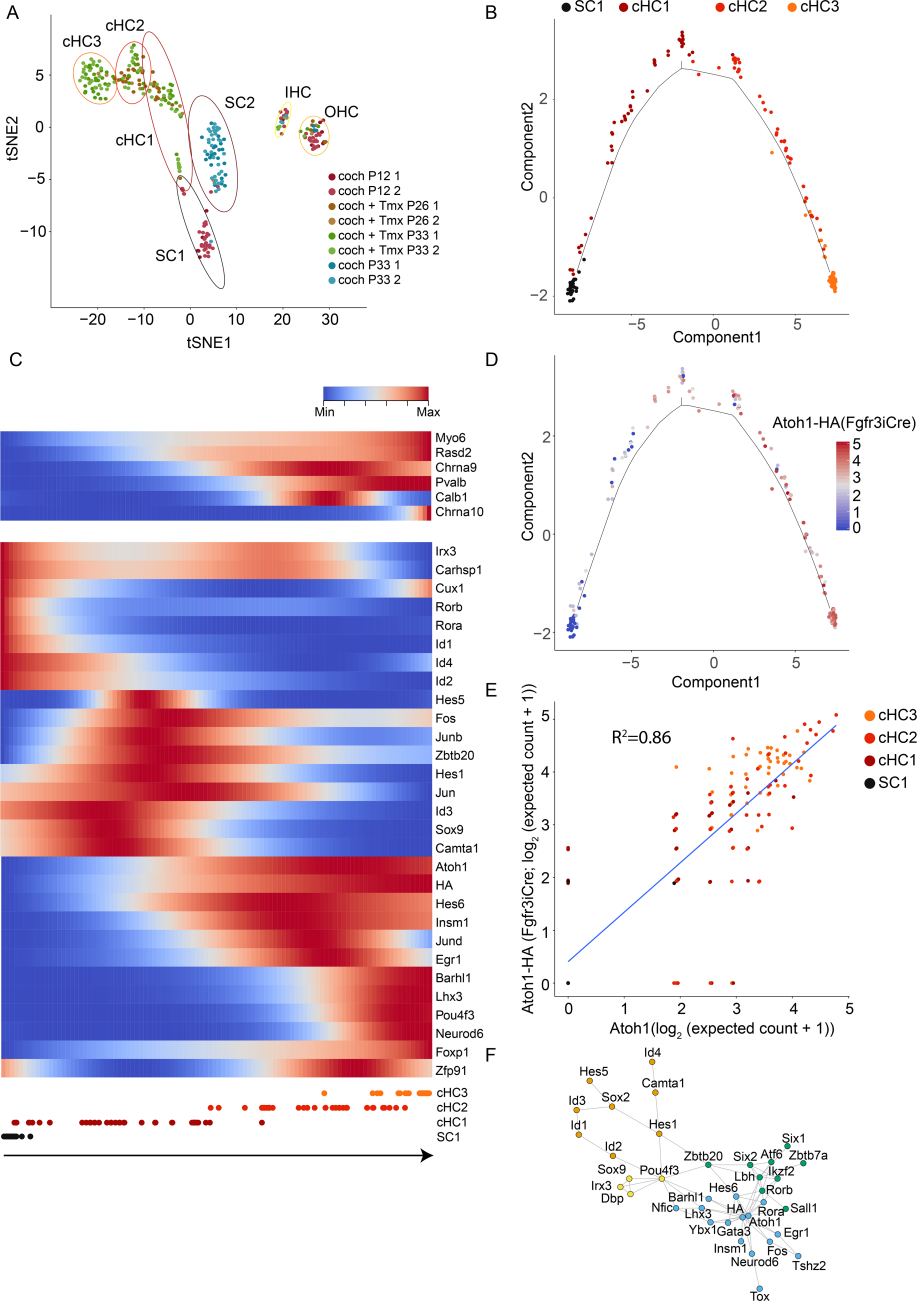
Atoh1-mediated SC-to-HC conversion is a continuum. (A) High-resolution map of SCs (DCs/PCs), cHCs, and HCs determined in Fig 1D. The HCs, SCs (DCs/PCs), and Atoh1+ cells were chosen and PCA followed by tSNE analyses were performed. Distinct colors were used for different conditions. Coch P12: cochlea at P12 with no tamoxifen-induction, Coch + Tmx P26: cochlea at P26 after tamoxifen-induction, Coch + Tmx P33: cochlea at P33 after tamoxifen induction, coch P33: cochlea at P33 with no tamoxifen induction. Each ellipse represents the 95% confident region for each cell type. (B) Pseudo-temporal ordering analysis. SC1 (black), cHC1 (dark red), cHC2 (vivid red), and cHC3 (vivid orange) cells were placed into two-dimensional space using Monocle (see methods). Black line indicates the conversion path. (C) Heatmap of HC marker and TF gene expression contributing to cell fate change from SC1 to cHC3 (lower panel) from red (maximum value for each gene in log_2_(expected count + 1) to blue (minimum value for each gene in log2(expected count + 1). Pseudotime was horizontally represented from left as a starting point to right as an end in 100 bins. (D) Atoh1-HA expression across pseudotime **Fig 2B**. (E) Correlation between Atoh1-HA and endogenous Atoh1 expression. R^2^ indicates the correlation coefficient. The units used were log_2_(expected count + 1). (F) Gene network analysis showing correlated TF genes. Expression levels of TF genes were extracted and Pearson’s correlations between them were calculated. Significantly correlated genes with R^2^ > 0.25 were connected by an edge. The shorter the line is, the stronger the correlation is while the longer the line is, the weaker the correlation is. Distinct groups determined by maximizing modularity Q [61] were indicated using distinct colors.

We next focused on cells undergoing conversion, which included the DC/PC (SC), cHC, and HC clusters. In total, we extracted 101 SCs, 60 HCs, and 145 cHCs from the full dataset. We then performed tSNE on this subset of the data, followed by shared nearest neighbor modularity optimization based clustering, which identified 7 unique clusters (**Fig 2A, S2 Fig and S2 Table; Figure Legends**).

To further validate our approach, we found markers were restricted to the expected clusters. For example, expression of *Fgfr3* and *Slc17a8* was restricted to the SCs and IHCs, respectively (**S2 Fig**). The SCs also separated into two clusters, with almost all SC1 cells coming from P12 cochleae while almost all SC2 cells were isolated from P26-33 cochleae **(Fig 2A, S1A** and **S2 Figs**). As Atoh1 induction occurs at P12, we used the SC1 cluster as the starting point for the induction of cHCs.

The cHCs separated into three distinct clusters (cHC1-3) (**Fig 2A**). Closer examination of marker expression in these cells found that canonical HC markers *Chrna10* and *Pou4f3* were almost uniquely expressed in cHC3, suggesting they are the most mature among cHCs and that there is a progression from SC1 to cHC1 to cHC2 to cHC3 [14, 21] (**Fig 2C** and **Fig 2A**). One important implication of this finding is that studying the previously unrecognized cHC1 and cHC2 that had undergone less complete conversion could identify factors that are needed to increase conversion efficiency.

To provide additional evidence that there is a continuum of cells present during HC conversion, we next ordered the cells in pseudotime with inverse graph embedding using Monocle2 [24]. The pseudotime reconstruction places individual cells in two-dimensional space in an unsupervised manner based on the relative transcription profiles of each cell. With this approach, the cells ordered along the anticipated trajectory of HC conversion starting with SC1 to cHC1 to cHC2 to cHC3 (**Fig 2B**). While transitioning from cHC1-2 to cHC3, canonical HC markers (*Myo6*, *Rasd2*, *Chrna9*, *Pvalb*, *Pou4f3*, *Chrna10*) started to be expressed (**Fig 2C**). Thus, inverse graph embedding of SCs undergoing Atoh1-mediated conversion further supported a continuum from a donor SC to a target state that resembled HCs.

To identify additional TFs that may be required to increase the efficiency and completion of Atoh1-mediated HC conversion, we plotted the expression of TFs that were found to be nonrandomly expressed across pseudotime using the beaming algorithm imbedded in Monocle2 (**Fig 2C**). As expected, TFs associated with the terminal differentiation of SCs, such as Rorb, Rora, Id1, Id4, Id2 [25, 26], decreased in expression as cells began undergoing Atoh1-mediated conversion. There was then a second cluster of genes that transiently increased in expression in cHC1, such as Hes5, Hes1, Sox9, Zbtb20 and members of the AP-1 complex (Fos, Junb, Jun). Subsequently, the cHC2 cluster was enriched with TFs such as Hes6, Insm1, Jund and Egr1, in addition to endogenous Atoh1 and exogenous Atoh1-HA. Finally, the cHC3 cluster highly expressed known HC TFs such as Barhl1, Lhx3, Pou4f3 and Neurod6 [27–30].

It has been shown that the expression of endogenous Atoh1 is upregulated during naturally occurring HC conversion in zebrafish and birds [31–34]. In our data, endogenous Atoh1 increases as conversion progresses (**S2A Fig**); transgenic Atoh1-HA expression strongly correlates with converted HC state (**Fig 2D** and **S2A Fig**), with cHC3 expressing 3.29 fold more Atoh1-HA than cHC1. This is unexpected because transgenic Atoh1-HA is driven by CAG promoter in Cre-positive cells, which should presumably have ubiquitously constant levels of transcriptional activity. Moreover, quantification of endogenous Atoh1 using the 3'UTR and exogenous Atoh1-HA using the HA tag, we found a strong correlation between the expression of both genes in each cell (R^2^ = 0.86; **Fig 2E**). These findings suggest there is a connection between endogenous Atoh1 and exogenous Atoh1-HA expression that is variable between cells but correlates strongly with the extent of conversion; the underlying mechanisms remain to be further studied.

To understand correlations between these TFs during the conversion continuum, we performed gene network analysis of TFs identified in our single-cell RNA-seq that were expressed above a threshold (i.e., detected in at least 10 cells) to remove noisy genes, and that also had high variance in expression across cells (variance >0.4) (**Fig 2F**). We identified four groups of TFs that showed high correlation within each group. As expected, Atoh1 was a central node to a large number of TFs (i.e., Barhl1, Lhx3, Gata3, Hes6, and Neurod6); Pou4f3 expression was also correlated to a distinct large group of TFs (i.e., Barhl1, Lhx3, Hes1, and Zbtb20). Therefore, such gene network analysis placed Atoh1 and Pou4f3 as key reprogramming factors for SC-to-HC conversion, which is supported by in vivo studies demonstrating that Pou4f3 synergistically induces Atoh1-mediated conversion or can promote conversion on its own [21].

### Bulk RNA-seq validation of isolated SCs, cHCs, and OHCs from mature cochleae

Single-cell RNA-seq provides high-resolution readouts of transcription within a tissue. However, this comes with the tradeoff of having limited sensitivity in a given cell where only 5–10% of transcripts are captured. This limit of sensitivity is especially problematic for genes that are expressed at low levels. To validate TFs that were found to be differentially expressed between SCs, cHCs, and OHCs in the single-cell RNA-seq data, we manually isolated, with high purity, individual cells of the three cell types based on marker expression and performed RNA-seq after whole-transcriptome amplification (**S3 Table**). These data were then compared to the single-cell RNA-seq to identify TFs consistently differentially expressed.

Specifically, after enzymatic dissociation of mouse cochleae, SCs (DCs and PCs) were isolated at P26 based on expression of Cre-positive tdTomato reporter, cHCs at P33 based on expression of both tdTomato and HC marker Chrna9-GFP, and OHCs at P7 or P22 based on expression of OHC marker Slc26a5 (**S3A–S3D Fig**). We also isolated IHCs at P74 (**S3B–S3F Fig**). A total of 10 bulk RNA-seq profiles (duplicates of five cell types) were produced, detecting expression of 23,415 unique genes in at least one sample (**S3C and S3D Fig**). The independent amplified bulk RNA-seq profiles of biological duplicates for each cell type were reproducible with a Spearman correlation of 0.86–0.89 (**S3E and S3F Fig**). In comparison, previous studies using replicates of RNA-seq data from samples that had undergone whole-transcriptome amplifications reported a Spearman correlation between biological duplicates of 0.8 [35]. To further validate the genes we found to be differentially expressed at the protein level, we found that 24 genes that had previously been shown to be differentially expressed in SCs, cHCs, and OHCs by immunostaining showed consistent expression patterns among SCs, cHCs and OHCs [14], and such patterns were also comparable to gene expression profiles determined by single-cell RNA-seq (**S3G Fig**).

To determine whether cHCs (P33) resembled differentiating neonatal HCs, as indicated by immunostaining and morphological results in previous reports [14, 21], we estimated, based on Spearman correlation analysis of bulk RNA-seq profiles, the distances between SCs (P26), OHCs (P7), OHCs (P22), IHCs (P74), and cHCs (P33). The distance of cHCs from OHCs (P7) was smaller than that from SCs, OHCs (P22), or IHCs (**S3H Fig**). These analyses provided evidence that cHCs resembled OHCs (P7) more than the mature SCs, and mature OHCs/IHCs analyzed. Cell type marker expression in SCs, cHCs, OHCs (P7), OHCs (P22), and IHCs was comparable to that observed using single-cell RNA-seq (**S3G Fig**).

**Fig 3:**
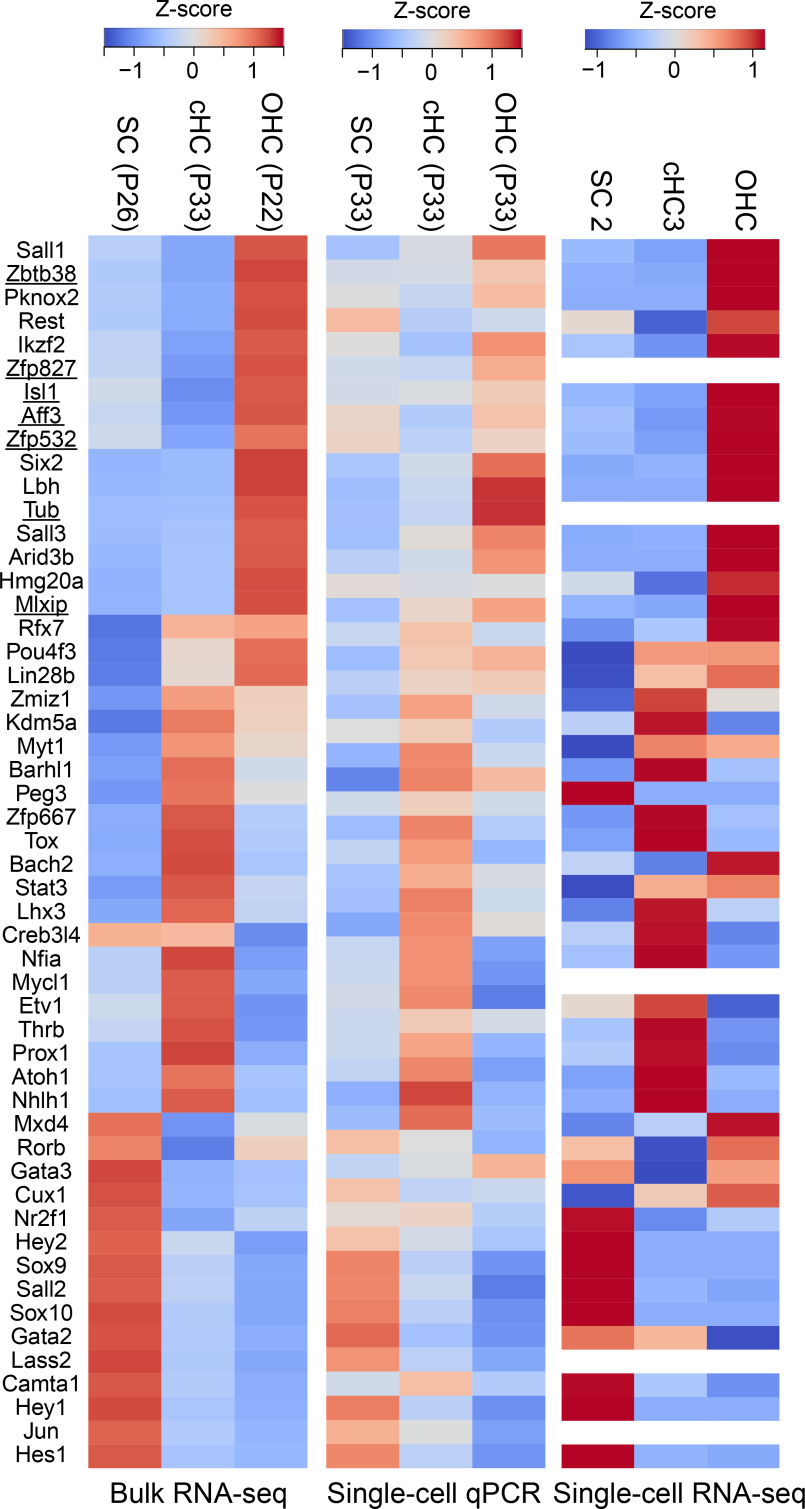
Differentially expressed TFs in SCs, cHCs, and OHCs. Heatmaps showing gene expression levels detected by bulk RNA-seq (left heatmap), single-cell qPCR (middle heatmap), and single-cell RNA-seq (right heatmap) for 52 differentially expressed TFs. *Isl1* and its six downstream target TFs are underlined [43].

To identify TFs that may promote Atoh1-mediated SC-to-HC conversion in vivo, we focused on TFs that are differentially expressed between mature SCs (P26), cHCs (P33), and mature OHCs (P22). Among 1,425 TFs in the mouse genome (Animal Transcription Factor Database, http://www.bioguo.org/AnimalTFDB/), we identified 90 TF genes that were differentially expressed, with statistical significance, in SCs (P26), cHCs (P33), and OHCs (P22) (**Fig 3, S4 Table**). To confirm that these TFs represented the underlying conversion of SCs to cHCs, we performed GO enrichment analysis where the most significantly differentially expressed categories included “sensory perception of sound”, “sensory perception of mechanical stimulus”, “neuron development”, “cell projection organization”, “inner ear development”, “sensory organ development”, and “synaptic transmission” (FDR < 0.05).

### Further validation with single-cell multiplex RT-qPCR of cochleae overexpressing Atoh1

We then utilized an orthogonal strategy to validate our list of differentially expressed genes by performing single-cell multiplex RT-qPCR analysis of 89 genes in manually isolated SCs, cHCs, and OHCs. These genes included: 1) 52 TF genes that we had identified as differentially expressed between SCs, cHCs, and OHCs in both single-cell and bulk RNA-seq; 2) specific SC or HC marker genes (i.e., *Slc26a5*, *Ocm*, *Gfi*, *Fgfr3*, *Cdkn1b*, *Gjb2*); and 3) housekeeping genes (i.e., *Gapdh*, *Actb*) to serve as controls (**S3I–S3M** and **S3P Fig**). Independent sets of single cells that included 27 SCs, 25 cHCs, and 16 OHCs, were isolated after enzymatic cochlear dissociation at P33 and analyzed with the Fluidigm BioMark high-throughput qPCR system (**S3I–S3M** and **S3P Fig**).

Comparison of the expression of TFs in the single-cell RNA-seq, bulk RNA-seq, and single-cell RT-qPCR datasets revealed a striking concordance among the three experimental strategies (**Fig 3**). To identify candidate TF genes that are required for transition from the SC/cHC1/2 states to cHC3 and whose overexpression could potentially increase conversion efficiency, we first focused on TFs overexpressed in cHC3 compared to SCs. As mentioned above, the cHCs (P33) manually isolated based on HC marker expression for bulk RNA-seq and single-cell qPCR are likely drawn from cHC2 and cHC3 in the single-cell RNA-seq samples. This approach revealed several TFs that could potentially increase the efficiency of conversion, including Barhl1, Lhx3, Nfia, and Pou4f3, many of which were also highly expressed in cHC3 cells in our single-cell RNA-seq analysis (**Fig 2C** and **S2A Fig**). As mentioned before, Pou4f3 has been shown to improve Atoh1-mediated conversion in vivo [21]. We also focused on TFs that are overexpressed in OHCs compared to cHC3 or cHCs (P33) whose overexpression could promote the completion of conversion; such TFs included Sall1/3, Ikzf2, Isl1, and Aff3 (**Fig 3**).

### Identification and validation of Isl1 as a potentiating co-reprogramming factor for Atoh1-mediated SC-to-HC conversion both ex vivo and in vivo

We next sought to identify the TFs that would be most likely to potentiate Atoh1-mediated SC-to-HC conversion. To accomplish this, we examined the list of genes overexpressed in OHCs for a TF that has been shown to regulate other differentially expressed genes. Strikingly, Isl1 was previously shown to regulate the expression of six of the 16 other overexpressed TFs, including *Mlxip*, *Zbtb38*, *Aff3*, *Zfp827*, and *Zfp532* in cardiac pacemaker cells [36] and *Tub* in retinal cells [37].

**Fig 4:**
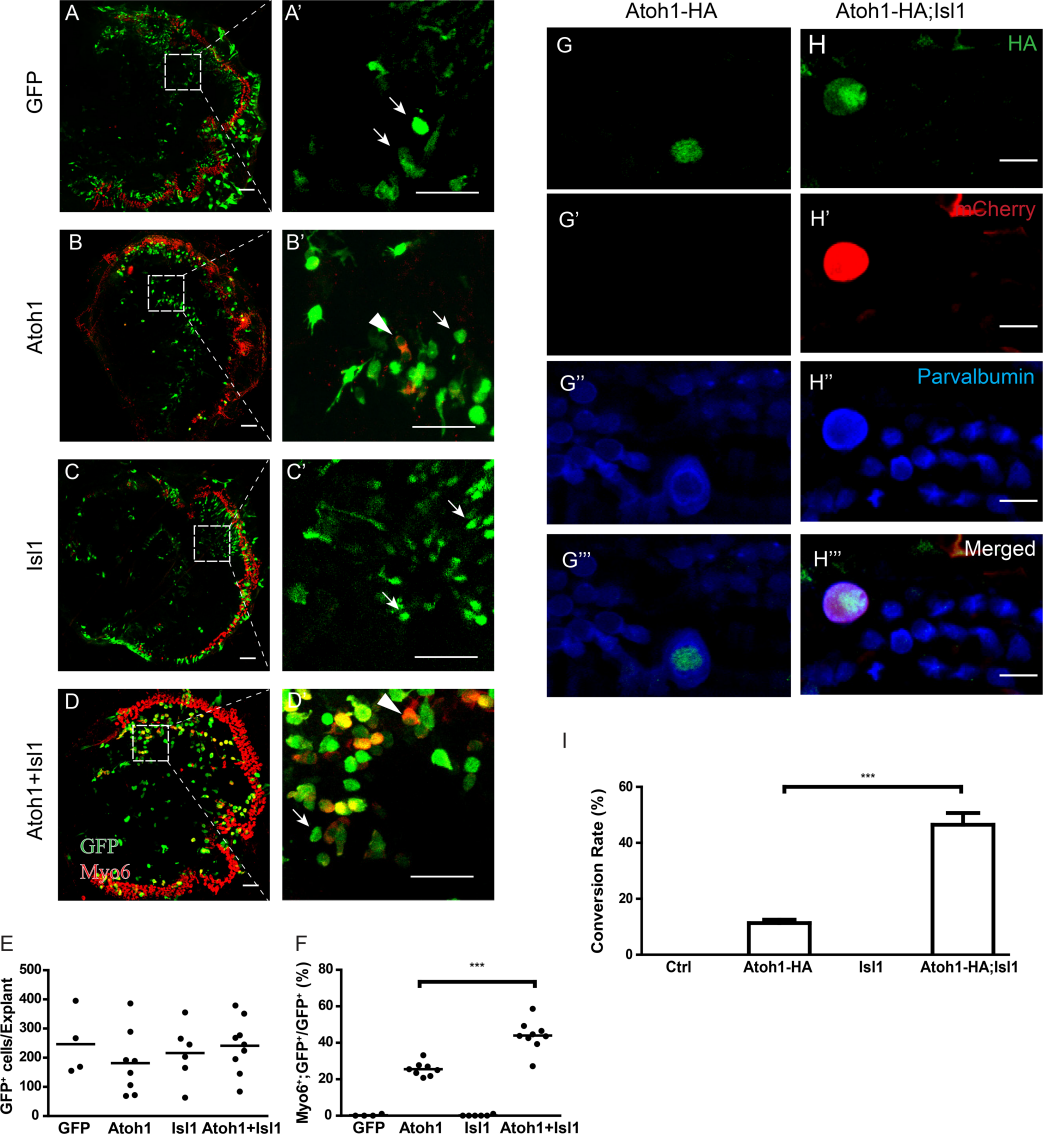
Isl1 synergistically enhances Atoh1-mediated SC-to-HC conversion *ex vivo* and *in vivo*. (A-D) Immunofluorescence staining of GFP (green) and Myo6 (red) in cultured cochlear explants 7 days after transfection. The explants were prepared from mice at P0 and transfected by electroporation with IRES-GFP alone (A and A’), Atoh1-IRES-GFP (Atoh1, B and B’), Isl1-IRES-GFP (Isl1, C and C’), or Atoh1-IRES-GFP and Isl1-IRES-GFP (Atoh1+Isl1, D and D’). GFP+ cells indicate transfected cells, and Myo6a is a HC marker. White arrows indicate nonconverted GFP-transfected GER cells, and white arrowheads indicate converted HCs that are positive for GFP and Myo6. The white boxes in panels A-D are enlarged in panels A’-D’, respectively. Scale bars represent 100 μm in A-D and 50 μm in A’-D’. (E, F) Scatter plots showing the conversion rates (E) as defined by the number of GFP+/Myo6+ double-positive cells divided by the number of GFP+ cells and the transfection rates (F) as defined by the number of GFP+ GER cells of corresponding explants. Each dot represents a sample; lines indicate mean values. ***P < 0.001 by Student’s t-test. (G-H) Immunofluorescence staining of HA (green), mCherry (red), and parvalbumin (blue) in the HC layer of cochlear wholemounts of Fgfr3-iCreER; Atoh1-HA (Atoh1-HA, G-G”‘) and Fgfr3-iCreER; Atoh1-HA; Isl1-IRES-mCherry (Atoh1-HA; Isl1, H-H”‘) mice. The mice that received tamoxifen injection at P12 and P13 were analyzed at P33. Scale bars represent 10 μm. (I) Quantification of conversion rate (see Methods for the calculation) in Fgfr3-iCreER; tdTomato (Ctrl), Atoh1-HA, Fgfr3-iCreER; Isl1-IRES-mCherry (Isl1), and Atoh1-HA; Isl1 mice. Data are presented as average ± SEM. ***P < 0.001 by Student’s t-test.

To determine if Isl1 can cooperate with Atoh1 to increase the SC-to-HC conversion efficiency and completion, we first overexpressed Isl1 in neonatal mouse cochlear explants. These ex vivo models have been shown to be surrogates of in vivo cochlear SC-to-HC conversion where cells analogous to SCs in the medial region (the greater epithelial ridge [GER]) of the cochlear explant can be converted to cHCs by ectopic Atoh1 expression [15, 38]. After transfecting the explants with empty (GFP) vector, *Atoh1*, *Isl1*, or both *Atoh1* and *Isl1*, we found a significant increase in the number of transfected GER cells converted into Myo6-expressing cHCs when both *Atoh1* and *Isl1* were transfected, whereas *Isl1* itself did not convert the GER cells to cHCs (**Fig 4A–4D**). The increase in the conversion rate in *Atoh1-Isl1* co-transfected cochleae was nearly double that in cochleae transfected with *Atoh1* alone (43.9% vs. 25.5%, 2-way ANOVA, p < 0.05), while the transfection rates among the four groups were similar (**Fig 4E and 4F**). We observed no significant difference in Ki67 staining among transfected GER cells (GFP+) between explants transfected with *Atoh1* alone and those transfected with *Atoh1+Isl1* (1.3% vs. 1.1%), indicating no significant proliferation was induced by the co-transfection.

To validate Isl1’s function in vivo, we created transgenic mouse lines to ectopically express Isl1 and to test whether the Isl1 co-expression in SCs synergistically enhances Atoh1-mediated conversion in vivo **(Fig 4H** and **S4 Fig)**. We inserted the CAG-loxp-stop-loxp-Isl1-IRES-mCherry fragment (which has the same backbone as the Atoh1-HA transgenic construct) into the mouse genome, and bred transgenic founders with the Fgfr3-iCreER mice to test Isl1 overexpression alone in PCs and DCs. In addition, we bred Isl1 transgenic founders with Fgfr3-iCreER and Atoh1-HA overexpressing mice to test the synergistic effect between Atoh1-HA and Isl1 in PCs and DCs. We used the same tamoxifen-mediated induction strategy for Atoh1- or Isl1-overexpression mice in PCs and DCs.

Similar to our ex vivo explant model, in two independent transgenic founder lines, co-expressing Isl1 with Atoh1 in SCs significantly increased the conversion rate compared with Atoh1 alone (50% vs 13%, see Materials and Methods for conversion rate calculation) (**Fig 4H–4J**) three weeks after tamoxifen induction (at P12). Expression of Isl1 alone, in contrast, failed to promote any conversion from SCs to HCs. Together, these *ex vivo* and *in vivo* results demonstrate that Isl1 synergistically enhances Atoh1-mediated SC-to-HC conversion by increasing the conversion rate. Moreover, the results validate our bulk, single-cell RNA-seq, and single-cell qPCR analyses, indicating that our approach can identify co-reprogramming factors that promote Atoh1-mediated SC-to-HC conversion.

## Discussion

In this study, we performed comprehensive, high-resolution transcriptional profiling of mouse organs of Corti after ectopic expression of Atoh1-HA in juvenile and adult SCs in vivo by using a combination of independent single-cell RNA-seq, bulk RNA-seq and single-cell multiplex qPCR analyses, all of which are consistent with each other. These analyses reveal a conversion continuum starting from initial donor SCs progressing towards a target state (cHC3) that most closely resembles neonatal differentiating HCs, while also uncovering unique early transition states (cHC1/2) of the conversion. Surprisingly, we found that expression of the transgene, Atoh1-HA, increased as the conversion progressed, and was highly correlated with that of endogenous Atoh1. Moreover, our approach identified 51 TF genes that are differentially expressed as cells undergo SC-to-HC conversion, and initiated a regulatory gene network of TFs that are required at different stages during HC reprograming. Importantly, we validated that one of these TFs, Isl1, synergistically promotes Atoh1-induced conversion *ex vivo* and *in vivo*.

### Single-cell transcriptomics reveals a continuum of SC-to-HC conversion by Atoh1

Based on our multi-faceted transcriptome profiling, our cHCs start with donor SCs (P12) when Atoh1-HA is ectopically expressed, gradually decrease expression of many SC-enriched genes, concomitantly gain HC signatures, and eventually reach the cHC3 state that closely resembles neonatal HCs (i.e., P7 OHCs). These results are consistent with our previous reports using morphological and immunostaining criteria [14, 21]. In >125 individual cHCs and >161 SCs/OHCs at three independent time points (P12, P26 and P33), the cHC conversion is a continuum, a conclusion also supported by our bulk RNA-seq and single-cell qPCR analyses. The pseudotemporal separation of the conversion enabled the logical identification of TFs that are required at different stages of conversion. Interestingly, we discover two clusters of early transition states (cHC1/2) that transiently express different sets of TF genes that can potentially contribute to the initial conversion from SCs at P12. By identifying differentially expressed TF genes between cHC3 and mature OHCs, we also list TFs that can presumably promote the completion of conversion. By gene network analysis, we discover the key TFs for conversion, including Atoh1, Pou4f3 and their closely correlated TFs, several of which have been previously demonstrated to contribute to SC-to-HC conversion [13, 14, 21, 39]. For example, Pou4f3 functionally promotes Atoh1-mediated conversion *in vitro* (together with Gfi1) [39] and *in vivo* [21], while GATA3 plays a key role in the Atoh1-mediated conversion in adult cochleae in vivo [21].

Future analyses of our high-resolution profiling can provide further insight into Atoh1-mediated conversion. For example, conversion paths are dependent on the starting donor cell’s genetic and epigenetic states, driving factors, and environment [2, 5, 40]. It is possible that distinct subpopulations of SCs provide the appropriate cellular context where Atoh1 has access to the required target loci to induce further conversion. Further work is needed to determine how closely early cHCs (i.e., cHC1/2) resemble otic progenitors, bypassing potentially harmful (i.e., tumorigenic) aspects of the progenitor phenotype.

### Progressively increased expression of endogenous and transgenic Atoh1 during conversion

Along with the revealed continuous process of conversion (from SC1 to cHC3), we also found that the expression of endogenous Atoh1 is progressively upregulated, with the lowest expression in the SC1 state and the highest expression in the cHC3 state. Such upregulation of endogenous Atoh1 is attributed to the ectopic expression of Atoh1-HA and the transcriptional autoregulation of the endogenous Atoh1 [31]. Interestingly, Atoh1-HA is also progressively upregulated during conversion from SC1 to cHC3. In fact, endogenous Atoh1 and transgenic Atoh1-HA were highly correlated in >125 cHCs analyzed. It is commonly assumed that the CAG promoter drives constant high levels of gene expression in all cells at all times; however, this may not be true in the organ of Corti. In support of this, tdTomato reporter expression driven by the CAG promoter at the ROSA locus also exhibits strikingly different intensities between IHCs and OHCs at P76 (**S3B Fig**). It is possible that both the CAG promoter for Atoh1-HA and the endogenous Atoh1 promoter are subject to transcriptional regulation by some common transcriptional or epigenetic factors [41]. It is also possible that Atoh1 and Atoh1-HA mRNAs are stabilized by common but unknown RNA binding proteins or miRNAs. Interestingly, Dicer mutant mice exhibited immature hair bundle morphology strikingly similar to that in Atoh1-HA-driven converted HCs [21, 42], linking miRNAs to Atoh1 mRNA stability.

### Additional transcription factors that promote Atoh1-mediated SC-to-HC conversion in vivo

For most direct reprogramming, multiple TFs function together, simultaneously or consecutively, either in the initiation stage or the later stage, to determine the cell fate and to induce efficient and complete conversion [2–5]. Thus, it is critical to identify TFs that improve Atoh1-mediated conversion. Here we have identified and validated 52 TF genes, including *Atoh1*, that are differentially expressed in SCs, cHCs, and OHCs. Notably, of these 52 TFs, Pou4f3 functionally promotes Atoh1-mediated conversion in vitro (together with Gfi1) [39] and in vivo [21] or by itself promotes conversion in vivo [21]. Interestingly, several TFs among the 52 are involved in development and cell fate reprogramming. For example, Isl1 is critical for pacemaker cell differentiation in the heart [43], and for motor neuron differentiation [44]. Several TFs (Isl1, Tub, Zbtb38, Zfp827, Aff3, Mixip and Zfp532) have been shown to be involved in pacemaker cell conversion in the heart [43]. Moreover, overexpression of Lhx3 in cochlear nonsensory cells is suggested to lead to Isl1 suppression [45]. Isl1 is also co-expressed with Atoh1 during early cochlear development [46]. Here we provided ex vivo and in vivo evidence that Isl1 indeed synergistically enhances Atoh1-mediated conversion in the cochlea. However, it remains unknown how Isl1 promotes Atoh1-mediated conversion. Notably, by forming complexes with different TFs (Lhx3 vs Phox2a), Isl1 can program ESCs to distinct cell identities (spinal vs. cranial motor neurons) [44, 47] by turning on variable groups of target genes determined by its binding partners. Thus, it will be important to identify the specific Isl1 binding complex in Atoh1-induced conversion. Moreover, future studies (e.g. single-cell RNA-seq and electrophysiology) on the Atoh1 and Isl1-converted HCs are also needed to molecularly and biologically characterize these cells to examine whether Isl1 also promotes HC maturation *in vivo*. Nonetheless, these results for Isl1 ex vivo and in vivo undoubtedly validate our single-cell transcriptomic analyses and identify 51 TFs that can promote Atoh1-mediated conversion in vivo.

SC-to-HC direct conversion is initially predominant in chicken HC regeneration that also starts with upregulation of Atoh1 in SCs [48] where the initial nuclear migration and other morphological cellular transformation are also similar to what we have characterized in our Atoh1-HA ectopic expression cochlear models [14, 21]. These parallels strengthen the notion that Atoh1-mediated HC conversion in the mature cochlea recapitulates the initial phases of naturally occurring HC conversion in non-mammalian species [48] and mammalian utricles [16].

Of note, our Atoh1-mediated SC-to-HC conversion path is remarkably similar to those reprogramming paths described in other regenerative systems. Ascl1-driven reprogramming of mouse embryonic fibroblast (MEF) cells to induced neurons (iN) in vitro exhibited a continuous conversion path nearly identical to the Atoh1-mediated conversion path [8]. Both Atoh1 and Ascl1 appear to act in a similar manner where donor cells overcome threshold barriers to initiate simultaneous upregulation of target cell fate genes and downregulation of donor cell fate genes. Moreover, in the MEF-to-iN conversion in vitro, additional reprogramming factors (Brn2 and Mytl1) further prevented competing myogenic programs and/or reversal to the initial donor state. While in our Atoh1-mediated SC-to-HC conversion, additional factors may play similar roles as Brn2 and Mytl1; the 51 TFs identified here may also benefit other regenerative systems by improving their efficiency and completion.

Together, our studies represent a major step towards understanding cochlear HC regeneration in vivo, with the potential to further improve the ongoing ATOH1 gene therapy in the clinic for patients with hearing loss. More importantly, our approach provides a valued strategy for better studying cochlea and other regenerative systems where conversion efficiency and completion are also central challenges.

## Materials and methods

### Animals

The Animal Care and Use Committee of St. Jude Children’s Research Hospital approved all protocols used in this study, and all methods were carried out in accordance with the approved guidelines. Mice were housed in a facility with a 12-h light/dark cycle and free access to food and water. Fgfr3^iCreER^, Atoh1-HA, Chrna9-EGFP, Ai14 and prestin-YFP mice were as described previously [22, 49–51]. Ai14 mice (termed here tdTomato mice) have a loxP-flanked stop cassette followed by a CAG promoter-driven red fluorescent protein variant (tdTomato) in the Rosa26 locus. The Pval-Cre mice were purchased from The Jackson Laboratory (Stock no. 008069). In order to label cHCs in vivo, tamoxifen was intraperitoneally injected into Fgfr3^iCreER+^; Atoh1-HA^+^; Chrna9-EGFP^+^; tdTomato^+^ mice at 3 mg/40 g at P12-13 (simplified as P12) as described before [14]. Fgfr3^iCreER+^; tdTomato^+^ mice were used to isolate SCs. We found strong tdTomato signals in IHCs but weak signals in OHCs in Pval-Cre^+^; tdTomato^+^ mice at P74 (**S3B Fig**). Therefore, Pval-Cre^+^; tdTomato^+^ were used to collect isolated IHCs.

### Bulk RNA-seq

Cochleae were dissected out, enzymatically digested, and triturated gently using fire-polished Pasteur pipettes. The enzymatic digestion was performed by incubating the cochleae in 1 mg/ml pronase (Roche life science) at 37°C for 40–50 min [52]. Total RNAs from handpicked fluorescently labeled isolated cells were purified as described previously [52]. The cDNAs were created and amplified using Single Primer Isothermal Amplification (SPIA) technology according to the manufacturer's instructions (NuGEN, San Carlos, CA). Libraries for RNA sequencing were generated with Encore NGS Library Systems according to manufacturer's instructions (NuGEN). The 100-bp paired-end reads were generated using an Illumina HiSeq 2000 system (Illumina, San Diego, CA). Base calling was performed using Illumina Casava 1.7. FASTQ adapter sequences were trimmed (with Cutadapt) and mapped to the mouse mm9 genome by a pipeline that serially employs STAR and BWA as described previously [53]. The mouse mm9 genomic sequence file was obtained from Genecode (https://www.gencodegenes.org/). The mapping statistics were determined using FlagStat in SAMtools [54] and the mapped reads were counted using HTSeq [55]. The count matrix was trimmed and mean of M-values (TMM)-normalized [56], and the differentially expressed genes were obtained using the limma-voom package in R [57, 58] after adjusting the p value for the false discovery rate (FDR) using Benjamini and Hochberg's adjustments [59]. To obtain differentially expressed transcription factors, gene sets encoding transcription factors/regulators from the Animal Transcription Factor Database (http://www.bioguo.org/AnimalTFDB/) were used. Fragments per kilobase of exon per million fragments mapped (FPKM) value for each gene was calculated by dividing the count of reads for each gene by total read counts for each sample, multiplied by 1,000,000, and divided by each transcript length in kb. The transcript length for each transcript was calculated by adding up the length of all exons annotated for each gene from the mm9 annotation file obtained from Genecode. All heatmaps represented in this study were drawn using the gplots package in R. In order to perform PCA analysis, count of reads were TMM-normalized and divided by the transcript length (TMM-normalized FPKM), and the numbers were used for subsequent PCA analysis. The PCA analysis was computed using the prcomp function in R after genes showing no expression in any cell type were removed. GO analysis was performed using DAVID Bioinformatics Resources 6.7 (https://david.ncifcrf.gov/). Only nonredundant GO terms were selected by using Revigo (http://revigo.irb.hr/) [60]. Spearman’s and Pearson’s correlations were computed using the cor function in R.

The RNA-seq data is available at Gene Expression Omnibus (GEO) submission: GSE85983 (NCBI tracking system #18023366).

### Single-cell qPCR

Total RNA (300ng) extracted from the inner ear of a C57BL6 mouse at P1 was converted into cDNA by using SuperScript VILO Master Mix (Thermo Fisher Scientific). After adding 2× TaqMan PreAmp Master Mix (Thermo Fisher Scientific) and 10× primer mix, pre-amplification of target genes was performed with 14 cycles of 95°C for 15 s and 60°C for 4 min. The cDNAs were treated with Exonuclease I (New England Biolabs, Beverley, MA), and the fivefold diluted cDNA was used to make a threefold dilution series of 15 concentrations. The samples were mixed with 2× SsoFast EvaGreen Supermix with Low Rox (Bio-Rad, Richmond, CA) and 20× DNA Binding Dye Sample Loading Reagent (Fluidigm, South San Francisco, CA). They were combined with 2× Assay Loading Reagent and pooled primer pairs in the 48.48 Dynamic Array integrated fluidic circuit (IFC) (Fluidigm) using the BioMark IFC controller MX (Fluidigm). The quantitative PCR was performed using a BioMark HD system (Fluidigm). The limit of detection threshold cycle (LOD-Ct) value was determined from the highest Ct value obtained using a threefold dilution series of 15 concentrations. The universal LOD-Ct was obtained by calculating the median value.

Handpicked fluorescent cells described above were lysed in 5 μL of lysis buffer containing 1× VILO reaction mix (Thermo), 1.2 U/L SUPERase•In RNase Inhibitor (Thermo Fisher Scientific), and 0.5% NP-40. The cDNA was generated in a 6-μL reaction volume after adding 0.15 μL of 10× SuperScript Enzyme Mix (Thermo Fisher Scientific) and 0.12 μL of T4 Gene 32 Protein (New England Biolabs). The RT reactions were performed as recommended by Fluidigm. Pre-amplification of target genes was performed, and the fivefold diluted cDNA was used for subsequent quantitative PCR analysis as described above and Cq value of each gene for each cell were obtained using Fluidigm Real-Time PCR Analysis software. The Cq values were converted to expression levels using the equation Log_2_(Ex) = (LOD-Ct) value—Cq.

### Single-cell RNA-seq

Cochleae were dissected out and incubated in solution (50% accutase (Innovative Cell Technologies, San Diego), 0.02% Trypsin(Thermofisher), 125 μg/ml Thermolysin (SIGMA-ALDRICH)) at 37°C for 3 min. 0.02 mg/ml collagenase IV (SIGMA-ALDRICH) and dispase (Worthington Biochemicals Corp) were further added to the enzyme solution and incubated for 4 min at 37°C. The enzymatically treated tissue was triturated gently using fire-polished Pasteur pipettes and aggregated cells were removed using a 40-μm strainer. The dissociated cells were centrifuged at 500 × g for 5 min and resuspended in solution (0.5% fetal bovine serum, 0.04% bovine serum albumin, 0.3 mM ethylenediaminetetraacetic acid in Hanks' balanced salt solution). Library construction was performed using Chromium Single Cell 3’ v2 Reagent Kits (10X genomics) following the instruction manuals. Briefly, the dissociated cells were loaded into a Chromium Controller (10X Genomics) and the encapsulated cells were lysed individually. Reverse transcription within each oil droplet was performed and the cDNAs were amplified. Next-generation sequencing was performed using an Illumina HiSeq 4000 system (Illumina, San Diego, CA) and the sequence reads were aligned to mouse reference genome mm10 using the Cell Ranger pipeline (10X Genomics). The obtained gene-barcode matrices were further analyzed using Seurat 1.4.0.16 in R. Briefly, cells with relatively small and large library size in individual datasets were individually removed as potential doublets and low-quality cells. Cell cycle classification was performed using scran 1.4.5 in R, and cells that were classified as being in G1 phase were chosen. To minimize batch effects, expected counts were obtained by randomly generating numbers following a binominal distribution with the minimum number of unique molecular identifiers for all the cells as a size parameter and the population of each gene as the probability. Cell cycle-related, apoptosis-related, and ribosomal protein-encoded genes were removed. Highly variable genes were chosen by calculating the average expression and dispersion for each gene using the MeanVarPlot function in the Seurat package with default parameters [23]. Subsequent PCA followed by t-Distributed Stochastic Neighbor Embedding was performed. Differentially expressed genes were identified using the FindAllMarkers function in the Seurat package with a default condition and PCA followed by tSNE analysis were further performed using the differentially expressed genes. Pseudo-temporal ordering analysis was performed using Monocle 2.4.0 [24]. Briefly, the expected counts were loaded into the Monocle package with a lowerDetectionLimit of 0.5. Highly variable genes with ≥ 0.1 mean expression and ≥ 1.0 empirical dispersion for each gene were then chosen. The cell trajectories were then drawn using the remaining genes.

To create a machine learning classifier, 20% and 80% of cells from each cluster were chosen as the test and training datasets using the StratifiedShuffleSplit function from scikit-learn 0.9.0 in python 3.5.3. Expression values for each gene were transformed into the range between 0 and 1 using the MinMaxScaler function, PCA without whitening was performed using the training dataset, and the 30 principle components were chosen. Optimal parameters for the support vector machine with a radial basal function, such as the C and gamma parameters were determined using the GridSearch CV function with a default conditions (3-fold cross-validation). Predicted clusters for the remaining 20% of cells were determined using the predict function and the predicted clusters were compared to clusters determined by PCA followed by tSNE using the confusion_matrix function. The prediction accuracy of the remaining datasets (20%) using the classifier was 100%, thus validating the clusters identified by tSNE.

### Cochlear explant culture and electroporation

The organs of Corti from P0.5 FVB mice were dissected and electroporated as described previously [38]. Briefly, the organ of Corti was isolated with the basal hook region removed to allow for improved adhesion. The tissue was then transferred to 150 μL HBSS in the center of a Millipore filter membrane (30 mm–diameter culture plate insert; Millipore, Billerica, MA) with the sensory epithelium facing up. Excess HBSS was carefully removed, and 5 μL of plasmid (1 μg/μL) was immediately added on top of the tissue. The volumes and DNA concentrations for the transfection were kept constant for all experiments. The epithelium on the filter was then placed in the center of a dish electrode (anode, 2 mm–diameter flat round electrode; NEPA GENE catalog no. CUY700P2E). A cover electrode (cathode, 2 mm–diameter flat round electrode; NEPA GENE catalog no. CUY700P2L) was positioned above the epithelium. Two rectangular pulses were delivered (28 V, 30 ms duration, with a 970-ms interval) using the NEPA GENE CUY21EDIT Square Wave Electroporator. The organ of Corti was then left standing for 1 min, after which 1 mL of Opti-DMEM was added to the membrane. The explant was then divided in two on the filter membrane, and the apical and basal sections were transferred to separate 5-cm glass-bottom culture dishes (Mattek) coated with Matrigel (Corning). A 2-mL volume of pre-warmed culture medium (high-glucose DMEM, 10% fetal bovine serum, 20 ng/mL epidermal growth factor, 10 uL/mL N2 supplement, 50 μg/mL ampicillin) was then added to each dish, and the dishes were incubated at 37°C in 5% CO2 and 95% humidity for the duration of the culture.

### Transgenic mouse lines of Isl1 ectopic expression

The coding region of the murine *Isl1* gene was cloned using RT-PCR of mouse cochlear total RNA and inserted right before the IRES sequence of the pCAGGS-S-stop-IRES-mCherry vector [21]. The 8.0kb DNA fragments of CAG-flox-stop-flox-Isl1-mCherry were taken out using PvuI and SapI and injected into the zygotes [14]. The offspring of ten founders were analyzed and five of them exhibited specific expression of mCherry in DCs/PCs at cochleae at P33 and later stages after tamoxifen injection at P12-13 when bred with Fgfr3^iCreER+^. The obtained conditional transgenic mice showed no obvious abnormal cochlear morphology either with or without Isl1-induction in DCs/PCs. At least two independent lines (#4 and #18) phenocopied the synergistic effects of Isl1 and Atoh1 in this study. The analysis shown in this study is from #18.

### Immunofluorescence and quantification of hair cell conversion rate

The tissues were fixed in 4% paraformaldehyde for 15 minutes at room temperature (cultured explants) or for overnight at 4°C (P33 cochleae). After washing in PBS and, for P33 cochlea, decalcification in 120mM EDTA, the samples were blocked and permeabilized in blocking buffer (10% horse serum, 1% BSA, and 1% Triton X-100 in PBS) for 1h at room temperature. They were then incubated at 4°C overnight in primary antibody solution. Primary antibodies used are as follows: Chicken anti-GFP antibody (1:1000, Abcam), rabbit anti-myosin VI (1:500 Proteus Bioscience), rat anti-HA (1:75, SIGMA), mouse anti-parvalbumin (1:500, Sigma), and rabbit anti-mCherry (1:1000, Abcam). The tissues were incubated with 1:1000 diluted secondary antibodies for 2 hours at room temperature, washed with PBS, and then mounted for imaging using ProLong Gold Antifade Reagent (Life Technologies). All images were taken under a Zeiss Axiophot 2 microscope with an LSM710 confocal laser scanning image system (Carl Zeiss, Jena, Germany).

To analyze the transfected explant cochleae, GFP^+^ cells in the greater epithelial ridge (GER) region of the organ of Corti with no obvious abnormal shape were counted as transfected cells. Of the GFP^+^ cells, myosin VI^+^ (MyoVI^+^) / GFP^+^ cells were counted as converted hair cells whereas MyoVI^+^/ GFP^+^ cells within clusters containing cells with MyoVI^+^/ GFP^+^ and with MyoVI^+^ were considered dislodged endogenous cells. The ratio of GFP^+^/ MyoVI^+^ cells to all GFP^+^ cells was presented as the conversion rate.

To analyze cochleae from transgenic mice, 200-μm-long regions in the middle turn of the cochleae were chosen, as described previously [14]. HA^+^ or mCherry^+^ were ectopically expressed only in DCs and PCs in this study. The conversion rate was calculated as the percentage of Parvalbumin^+^/ HA^+^ cells in all HA^+^ cells (for all Atoh1-HA mice), Parvalbumin^+^/ mCherry^+^ cells in all mCherry^+^ cells (for all Isl1 mice), or parvalbumin^+^/ HA^+^/ mCherry^+^ cells in all HA^+^/ mCherry^+^ cells (for all Atoh1-HA; Isl1 mice).

## Supporting information

S1 Fig Validation of single-cell RNA-seq. (A) PCA followed by tSNE analysis for all 5470 cochlear cells. Distinct colors were used for different conditions. Coch P12: cochlea at P12 with no tamoxifen induction, Coch + Tmx P26: cochlea at P26 after tamoxifen induction, Coch + Tmx P33: cochlea at P33 after tamoxifen induction, coch P33: cochlea at P33 with no tamoxifen induction.
(B) PCA followed by tSNE analysis for all 5470 cochlear cells with mapping of expression levels of specific markers. The two-dimensional spaces shown correspond to that in S1A Fig. Slc26a5 and Ocm were highly expressed in mature OHCs (P22) but not in cHCs (P33) or SCs (P26) [14]. GATA3 proteins have been observed in IHCs, IPhs/IBs, and Hensen cells in mice at P30 [21]. Slc1a3 expression has been observed in IPhs/IBs in organs of Corti in adult mice [62]. Plp is specifically expressed in Schwann and satellite cells in adult mouse cochleae [63]. Mpz is known as a Schwann cell marker [64]. Strong Emilin2 mRNA expression has been specifically detected in the tympanic border cells underneath of basilar membrane in mice at P8 and P13 [65]. Specific mRNA expression of Vmo1 has been detected in Reissner membrane in mice at P5 [66]. βIII tubulin is known as a specific marker of type I spiral ganglion [67]. Fst is expressed in the lesser epithelial ridge in mouse cochleae at P8 [68]. Gjb2 is highly expressed in the outer sulcus region, as well as in DCs/PCs, Iphs/IBs and Hensen cells [69]. Col11a2 is expressed in spiral limbus region of mouse cochleae at P5 [70]. Cx3cr1 and Cd79a are pan-macrophage and B-cell markers, respectively [71, 72]. Nkg7 is known to be highly expressed in NKT1 cells [73].
(C) Proportion of each population present in each sample. The proportion of each population was calculated using the cell number for each cluster divided by the total number of cells in each sample (number on the right in each row). Different clusters are represented by different colors.
(PDF)

S2 Fig Expression of markers and TFs in SCs, cHCs, and HCs. (A) Higher-resolution map of SCs, cHCs, and HCs determined in Fig 2A with expression levels of cell type-specific markers.
(B) Fine-resolution map of SCs, cHCs, and HCs determined in Fig 2A with expression levels of TF genes obtained by gene network analysis in Fig 2F. The colors placed above the two-dimensional spaces correspond to those in Fig 2F. The expression level for each gene in A-B is color-coded from red (maximum) to blue (minimum) based on log_2_ (expected count + 1).
(PDF)

S3 Fig Validation of bulk RNA-seq and single-cell qPCR. (A) OHCs labeled with prestin-YFP (green) in prestin-YFP knock-in cochleae from mice at P21 [51]. Myo6 (red) labels the cytoplasm of both OHCs and IHCs, while prestin, encoded by *Slc26a5*, labels the plasma membrane of OHCs. Lower panels show an optical section in the dotted line of the cochlear wholemount.
(B) IHCs labeled with tdTomato (red) in pval-Cre^+^; Rosa26-CAG-loxP-stop-loxP-tdTomato^+^ cochleae from mice at P76. Myo6 (green) labels cytoplasm of both OHCs and IHCs. Lower panels show an optical section in the dotted line of the cochlear wholemount. Scale bars: 20 μm (for A-B).
(C) Summary of mouse lines, tamoxifen injection ages, and harvest ages of different cell types for bulk RNA-seq and single-cell qPCR.
(D) Mapping statistics (mean ± S.D.) of the RNA-seq data. Cell #: number of cells collected for each bulk RNA-seq profiling; read #: number of total reads obtained in millions; mapped%: percentage of mapped reads.
(E) Heatmap showing correlation coefficients among the five cell types analyzed with biological duplicates for each cell type. The Spearman’s correlations are color-coded from red (maximum, 1) to blue (minimum, 0.7). The correlation coefficients between different samples are also indicated. Note that correlation coefficients between biological duplicates of each cell type are 0.86–0.89.
(F) Heat map showing correlation coefficients among the five cell types using genes only for TFs. The Spearman’s correlations are color-coded from red (maximum, 1) to blue (minimum, 0.7). The correlation coefficients between different samples are also indicated.
(G) Heatmap showing the expression profiles for 24 known genes [14] in mature SCs, cHCs, and HCs based on bulk RNA-seq (left) and in SC2s, cHC3s, and HCs based on single-cell RNA-seq (right). The average levels of gene expression in mature SCs (P26), cHCs (P33), mature IHCs (P74), OHCs (P7), and mature OHCs (P22) using Z-scores after TMM-normalization are shown (left) while the average levels of gene expression (log_2_(expected count + 1)) in SC2s, cHC3s, OHCs, and IHCs using Z-scores are shown. The expression level is color-coded from red (maximum) to blue (minimum). Known OHC-, IHC-, HC- and SC-specific markers are indicated. Eleven HC-specific genes whose products were previously detected by immunostaining in cHCs [14] are underlined in red, while OHC-specific *Slc26a5* and *Ocm*, whose products were not previously detected in cHCs [14], are underlined in red dashed lines. Sox2, whose product was sporadically down-regulated by immunostaining in cHCs [14] is underlined in with red dashed lines.
(H) Heatmap showing distance from cHCs to SCs, P7 OHCs, mature OHCs, and IHCs. Spearman_all: The distance was obtained based on Spearman’s correlation including all genes. Spearman_TF: The distance was obtained based on Spearman’s correlation including gene expression of 1425 transcription factors. PCA_all: The distance was obtained based in PC space using all genes. PCA_TF: The distance was obtained based in PC space using the expression of 1425 TFs. PCA_MAD: The distance was obtained based in PC space using the median absolute deviation of expression of the top 200 genes. PCA_4 fold: The distance was obtained based in PC space using the expression of genes with a fourfold change between the different cell types.
(I) Heatmaps showing Ct values obtained using different PCR primer sets (columns) and serially diluted inner-ear cDNA obtained from mice at P1 (rows).
(J) Examples of melting curves obtained using the primer set to amplify the Atoh1 gene. Results with more than one peak were removed for analysis purposes.
(K) Histogram showing the PCR efficiency distribution for all primer sets used in this assay [shown in A)]. Values are given as the mean ± SEM.
(L) Quantile–quantile plot of log-transformed *Actb* expression determined by qPCR for SCs (top section), cHCs (middle section), and OHCs (bottom section).
(M) Violin plots showing the mRNA expression levels (log_2_(Ex)) of six representative genes in SCs (black), cHCs (dark red), and OHCs (portland orange). See plots of the remaining genes in **S3P Fig**. An approximation of frequency distribution (gray) was determined by kernel density estimation. Portland orange boxes indicate genes known to be expressed in mature OHCs, while black boxes indicate genes known to be expressed in mature SCs. Atoh1 and Pou4f3 are known to be up-regulated in cHCs compared to those in SCs, as we previously showed using immunostaining [14]. Values are the mean ± SD. *P < 0.05 by one-way ANOVA followed by Student's *t*-test with a Bonferroni correction.
(N and O) Correlation between bulk RNA-seq and single-cell qPCR results. The Log ratios of expression between cHCs and SCs (N) or between OHCs and cHCs (O) were determined by single-cell qPCR (x-axis) and bulk RNA-seq (y-axis). Each dot represents one of the 89 genes analyzed by both methods. The Pearson’s correlations (r^2^) and the linear regression lines (blue) are indicated.
(P) Violin plots showing the expression levels of genes tested by single-cell qPCR in SCs (black), cHCs (dark red), and OHCs (portland orange). An approximation of frequency distribution is indicated (gray), as determined by kernel density estimation. Note that the PCR results for STAT3 in 20 SCs were only included to draw violin plots because of technical difficulties with 7 out of 27 test chambers due to technical errors. Values are given as the mean ± SD. *P < 0.05 by two-way ANOVA followed by Student's *t*-test with a Bonferroni correction.
(Q) GO term enrichment for bulk RNA-sequencing experiments.
(PDF)

S4 Fig A schematic representation of transgene to ectopically express Isl1 and mCherry upon Cre-mediated deletion. (PDF)

S1 Table This table is associated with Fig 1E. The mean expression of the clustered cells used as input for the heatmap.
(XLSX)

S2 Table This table is associated with Fig 2. The raw counts are shown for each sample with a unique identification sequence.
(XLSX)

S3 Table This table is associated with Figs 3 and S3. The FPKM data were read-count and gene-length corrected and filtered for minimum expression. The CPM data corrected for read count, TMM-normalized and filtered for minimum expression used as input for heatmaps.
(XLSX)

S4 Table This table is associated with Figs 3 and S3. Transcription factor data is the subset of CPM TMM-normalized data for the transcription factor data set. The data for the 52 selected genes are the CPM TMM-normalized data for this select class.
(XLSX)
