# Supplementary figures and images for: High-resolution transcriptional dissection of in vivo Atoh1-mediated hair cell conversion in mature cochleae identifies Isl1 as a co-reprogramming factor
Source: PLoS Genet. 2018 Jul 31;14(7):e1007552. doi: 10.1371/journal.pgen.1007552 (PMC6086484; doi:10.1371/journal.pgen.1007552)

A

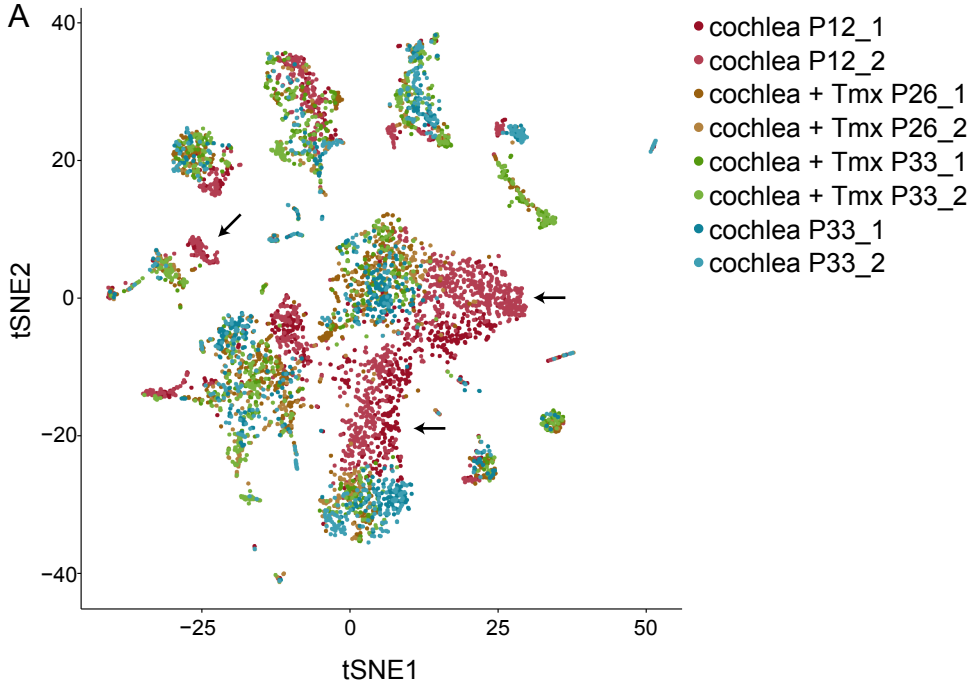

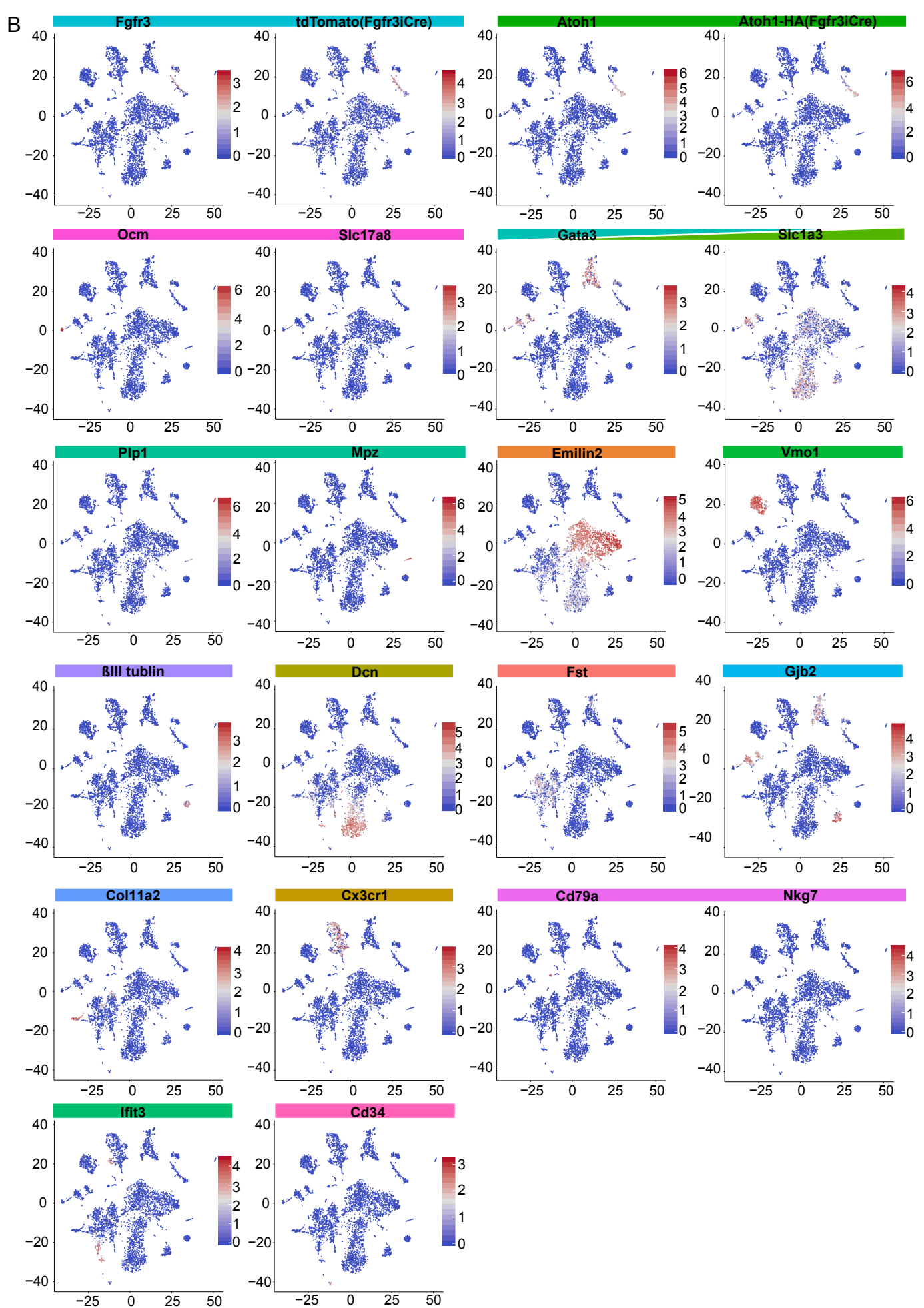

C

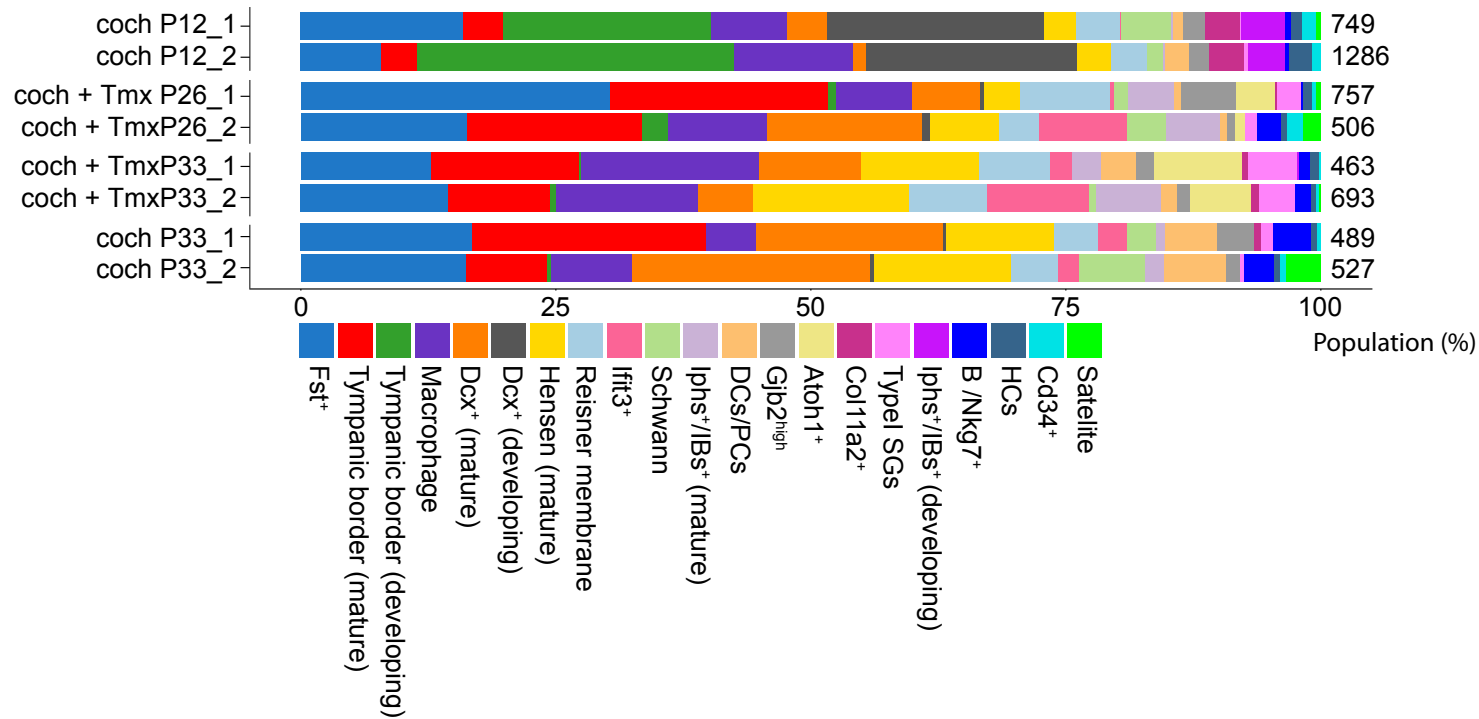

Supplement: S1 Fig — (A) PCA followed by tSNE analysis for all 5470 cochlear cells. Distinct colors were used for different conditions. Coch P12: cochlea at P12 with no tamoxifen induction, Coch + Tmx P26: cochlea at P26 after tamoxifen induction, Coch + Tmx P33: cochlea at P33 after tamoxifen induction, coch P33: cochlea at P33 with no tamoxifen induction. (B) PCA followed by tSNE analysis for all 5470 cochlear cells with mapping of expression levels of specific markers. The two-dimensional spaces shown correspond to that in S1A Fig. Slc26a5 and Ocm were highly expressed in mature OHCs (P22) but not in cHCs (P33) or SCs (P26) [14]. GATA3 proteins have been observed in IHCs, IPhs/IBs, and Hensen cells in mice at P30 [21]. Slc1a3 expression has been observed in IPhs/IBs in organs of Corti in adult mice [62]. Plp is specifically expressed in Schwann and satellite cells in adult mouse cochleae [63]. Mpz is known as a Schwann cell marker [64]. Strong Emilin2 mRNA expression has been specifically detected in the tympanic border cells underneath of basilar membrane in mice at P8 and P13 [65]. Specific mRNA expression of Vmo1 has been detected in Reissner membrane in mice at P5 [66]. βIII tubulin is known as a specific marker of type I spiral ganglion [67]. Fst is expressed in the lesser epithelial ridge in mouse cochleae at P8 [68]. Gjb2 is highly expressed in the outer sulcus region, as well as in DCs/PCs, Iphs/IBs and Hensen cells [69]. Col11a2 is expressed in spiral limbus region of mouse cochleae at P5 [70]. Cx3cr1 and Cd79a are pan-macrophage and B-cell markers, respectively [71, 72]. Nkg7 is known to be highly expressed in NKT1 cells [73]. (C) Proportion of each population present in each sample. The proportion of each population was calculated using the cell number for each cluster divided by the total number of cells in each sample (number on the right in each row). Different clusters are represented by different colors. (PDF) [file pgen.1007552.s001.pdf]

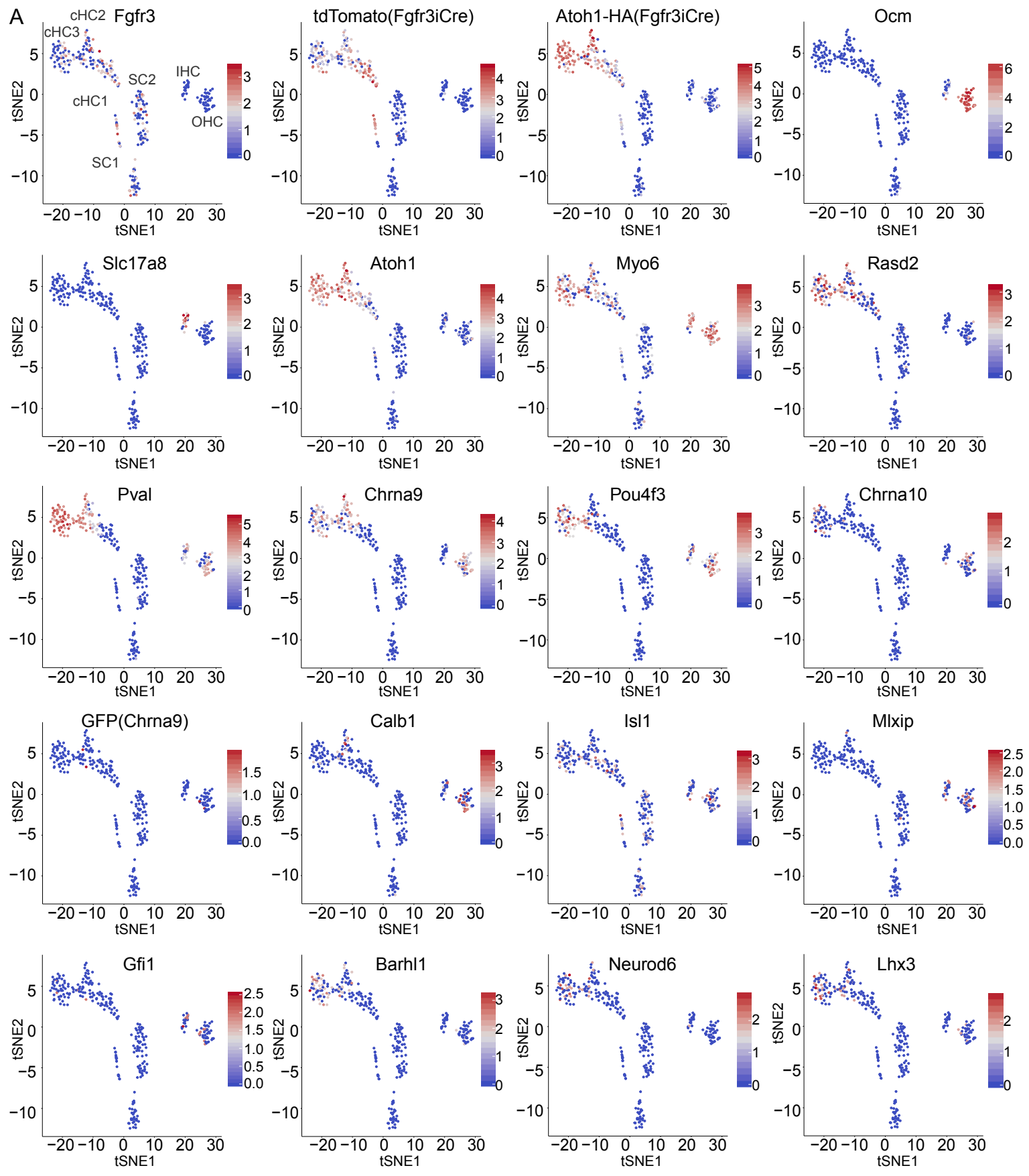

B

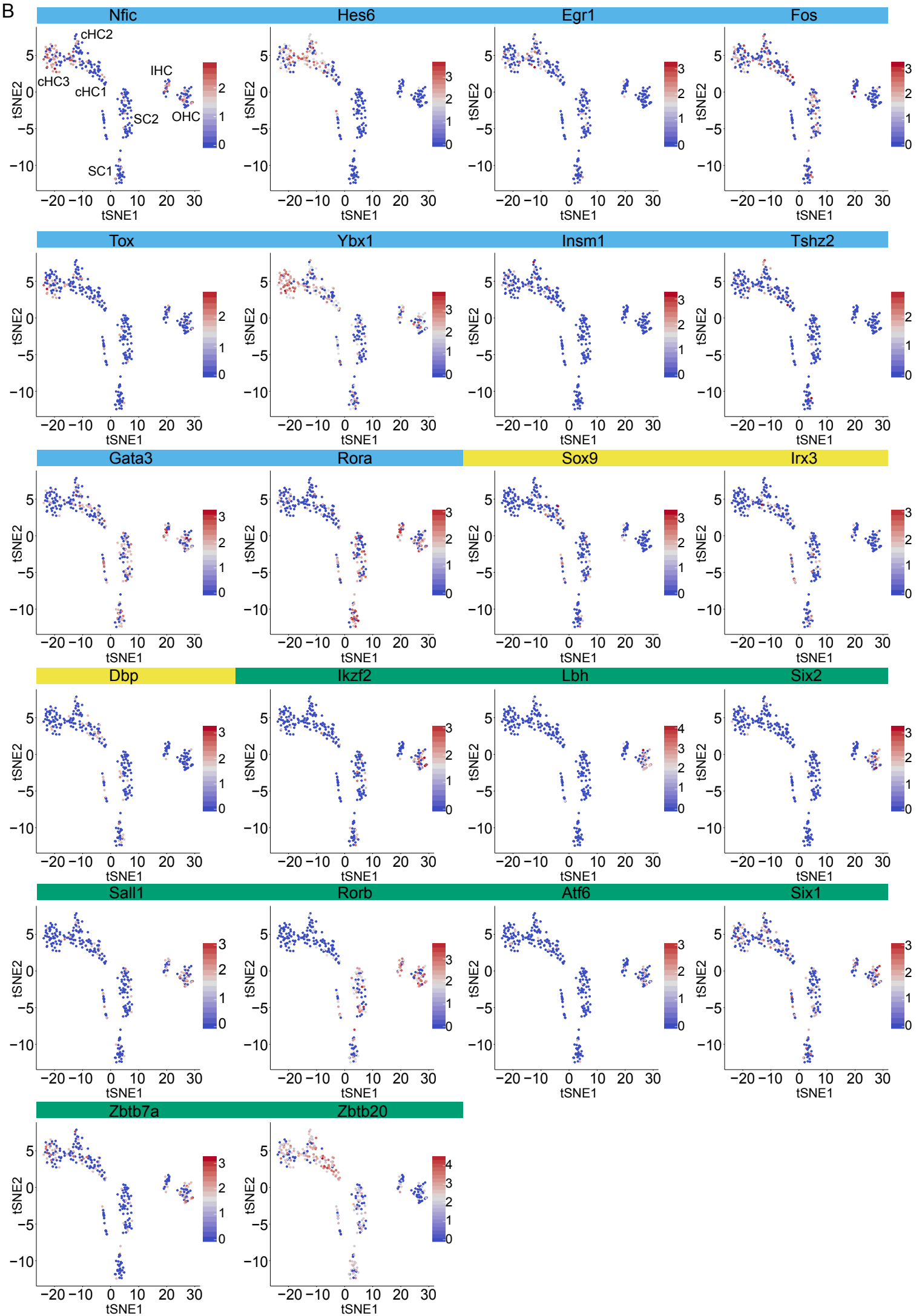

## B continued

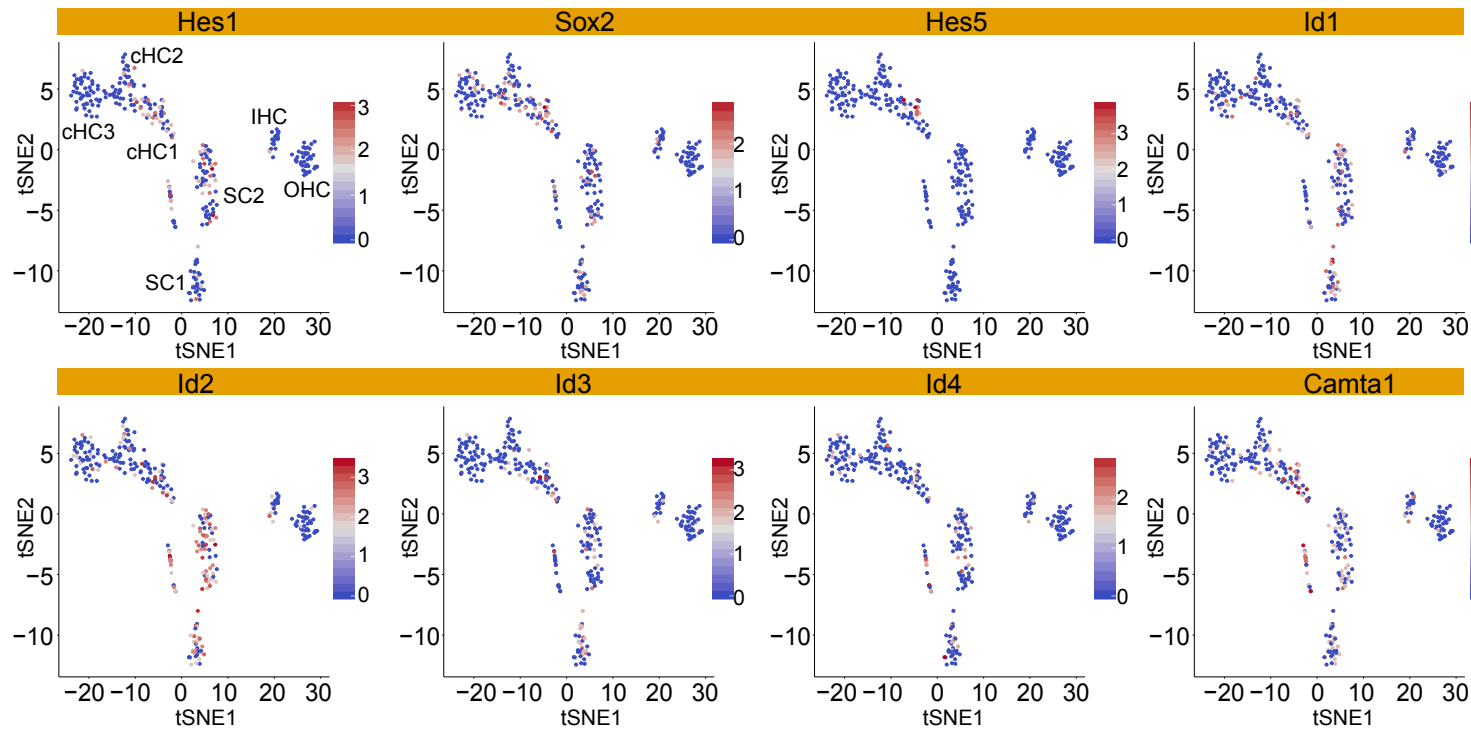

Supplement: S2 Fig — (A) Higher-resolution map of SCs, cHCs, and HCs determined in Fig 2A with expression levels of cell type-specific markers. (B) Fine-resolution map of SCs, cHCs, and HCs determined in Fig 2A with expression levels of TF genes obtained by gene network analysis in Fig 2F. The colors placed above the two-dimensional spaces correspond to those in Fig 2F. The expression level for each gene in A-B is color-coded from red (maximum) to blue (minimum) based on log2 (expected count + 1). (PDF) [file pgen.1007552.s002.pdf]

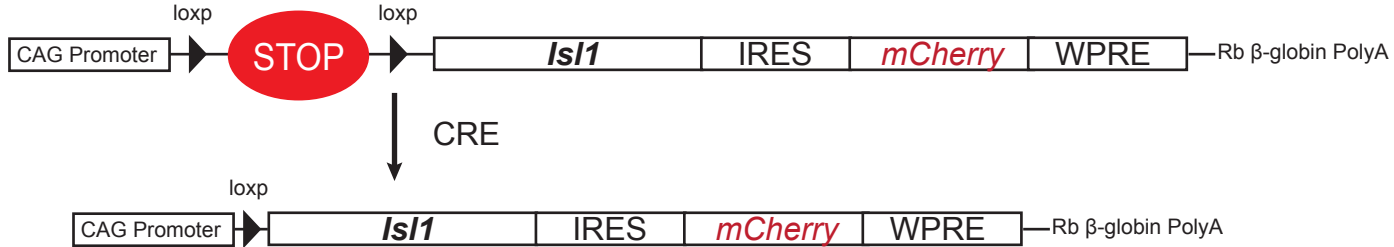

Supplement: S4 Fig — (PDF) [file pgen.1007552.s004.pdf]
