# Supplementary material for: High-resolution transcriptional dissection of in vivo Atoh1-mediated hair cell conversion in mature cochleae identifies Isl1 as a co-reprogramming factor
Source: PLoS Genet. 2018 Jul 31;14(7):e1007552. doi: 10.1371/journal.pgen.1007552 (PMC6086484; doi:10.1371/journal.pgen.1007552)
Supplement: S3 Fig — (A) OHCs labeled with prestin-YFP (green) in prestin-YFP knock-in cochleae from mice at P21 [51]. Myo6 (red) labels the cytoplasm of both OHCs and IHCs, while prestin, encoded by Slc26a5, labels the plasma membrane of OHCs. Lower panels show an optical section in the dotted line of the cochlear wholemount. (B) IHCs labeled with tdTomato (red) in pval-Cre+; Rosa26-CAG-loxP-stop-loxP-tdTomato+ cochleae from mice at P76. Myo6 (green) labels cytoplasm of both OHCs and IHCs. Lower panels show an optical section in the dotted line of the cochlear wholemount. Scale bars: 20 μm (for A-B). (C) Summary of mouse lines, tamoxifen injection ages, and harvest ages of different cell types for bulk RNA-seq and single-cell qPCR. (D) Mapping statistics (mean ± S.D.) of the RNA-seq data. Cell #: number of cells collected for each bulk RNA-seq profiling; read #: number of total reads obtained in millions; mapped%: percentage of mapped reads. (E) Heatmap showing correlation coefficients among the five cell types analyzed with biological duplicates for each cell type. The Spearman’s correlations are color-coded from red (maximum, 1) to blue (minimum, 0.7). The correlation coefficients between different samples are also indicated. Note that correlation coefficients between biological duplicates of each cell type are 0.86–0.89. (F) Heat map showing correlation coefficients among the five cell types using genes only for TFs. The Spearman’s correlations are color-coded from red (maximum, 1) to blue (minimum, 0.7). The correlation coefficients between different samples are also indicated. (G) Heatmap showing the expression profiles for 24 known genes [14] in mature SCs, cHCs, and HCs based on bulk RNA-seq (left) and in SC2s, cHC3s, and HCs based on single-cell RNA-seq (right). The average levels of gene expression in mature SCs (P26), cHCs (P33), mature IHCs (P74), OHCs (P7), and mature OHCs (P22) using Z-scores after TMM-normalization are shown (left) while the average levels of gene express [file pgen.1007552.s003.pdf]

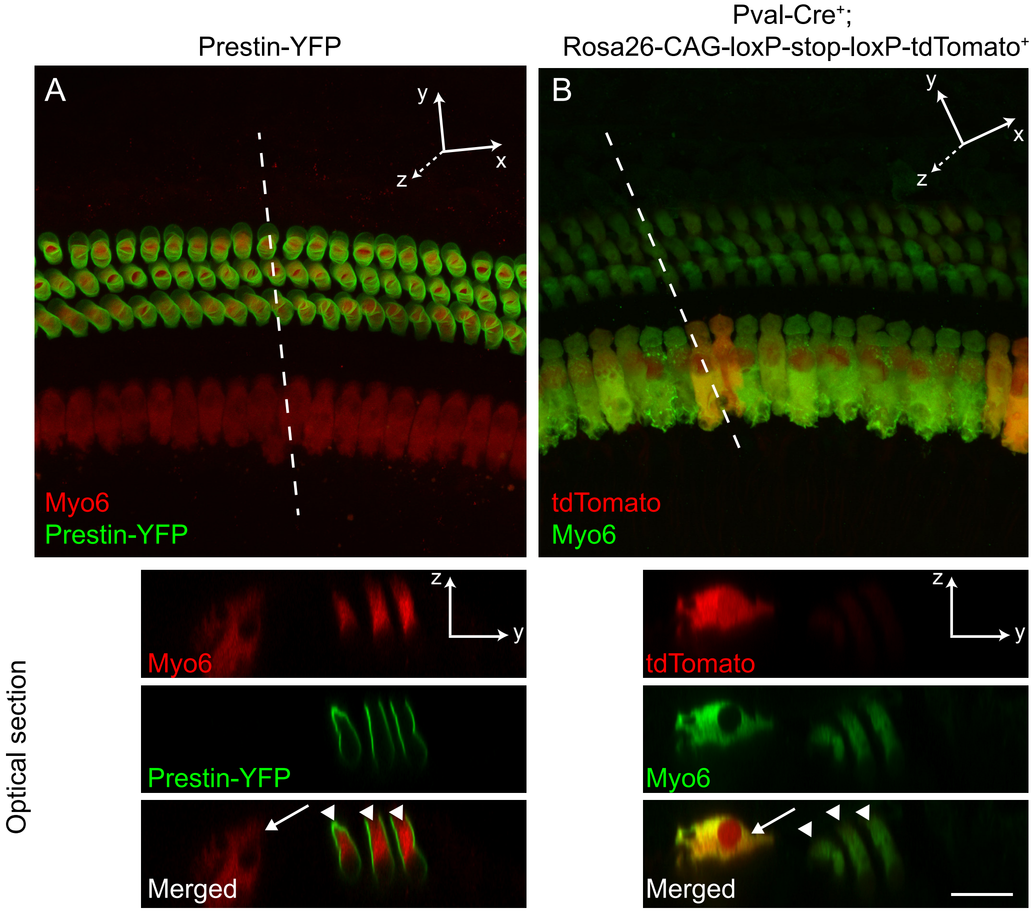


C

| Cell type | Mouse lines | Tamoxifen | Age (bulk RNA-Seq) | Age (sc-qPCR) |
|---|---|---|---|---|
| SC (P26) | Fgfr3iCreER$^{+}$; Rosa26-CAG-loxP-stop-loxP-tdTomato$^{+}$ | P12 & 13 | P26 & 27 | P33 |
| cHC (P33) | Fgfr3iCreER$^{+}$; Atoh1-HA$^{+}$; Chrna9-EGFP$^{+}$; Rosa26-CAG-loxP-stop-loxP-tdTomato$^{+}$ | P12 & 13 | P33 | P33 |
| OHC (P7) | PrestinCreER$^{T2+}$; Rosa26-CAG-loxP-stop-loxP-tdTomato$^{+}$ | P2 & 3 | P7 | N.A. |
| OHC (P22) | Prestin-YFP | N.A. | P22 & 23 | P33 |
| IHC (P74) | Pval-Cre$^{+}$; Rosa26-CAG-loxP-stop-loxP-tdTomato$^{+}$ | N.A. | P74 & 75 | N.A |

D

| Cell type | Cell# | Read # (× 10$^{6}$) | Mapped% |
|---|---|---|---|
| SC (P26) | 36.5 ± 5.5 | 118 ± 53 | 83.0 ± 0.1 |
| cHC (P33) | 22.5 ± 0.5 | 121 ± 43 | 84.7 ± 1.6 |
| OHC (P7) | 29.5 ± 6.5 | 100 ± 44 | 84.1 ± 0.0 |
| OHC (P22) | 21 ± 0 | 67 ± 39 | 79.6 ± 7.1 |
| IHC (P74) | 85 ± 46 | 67 ± 1 | 79.6 ± 0.2 |

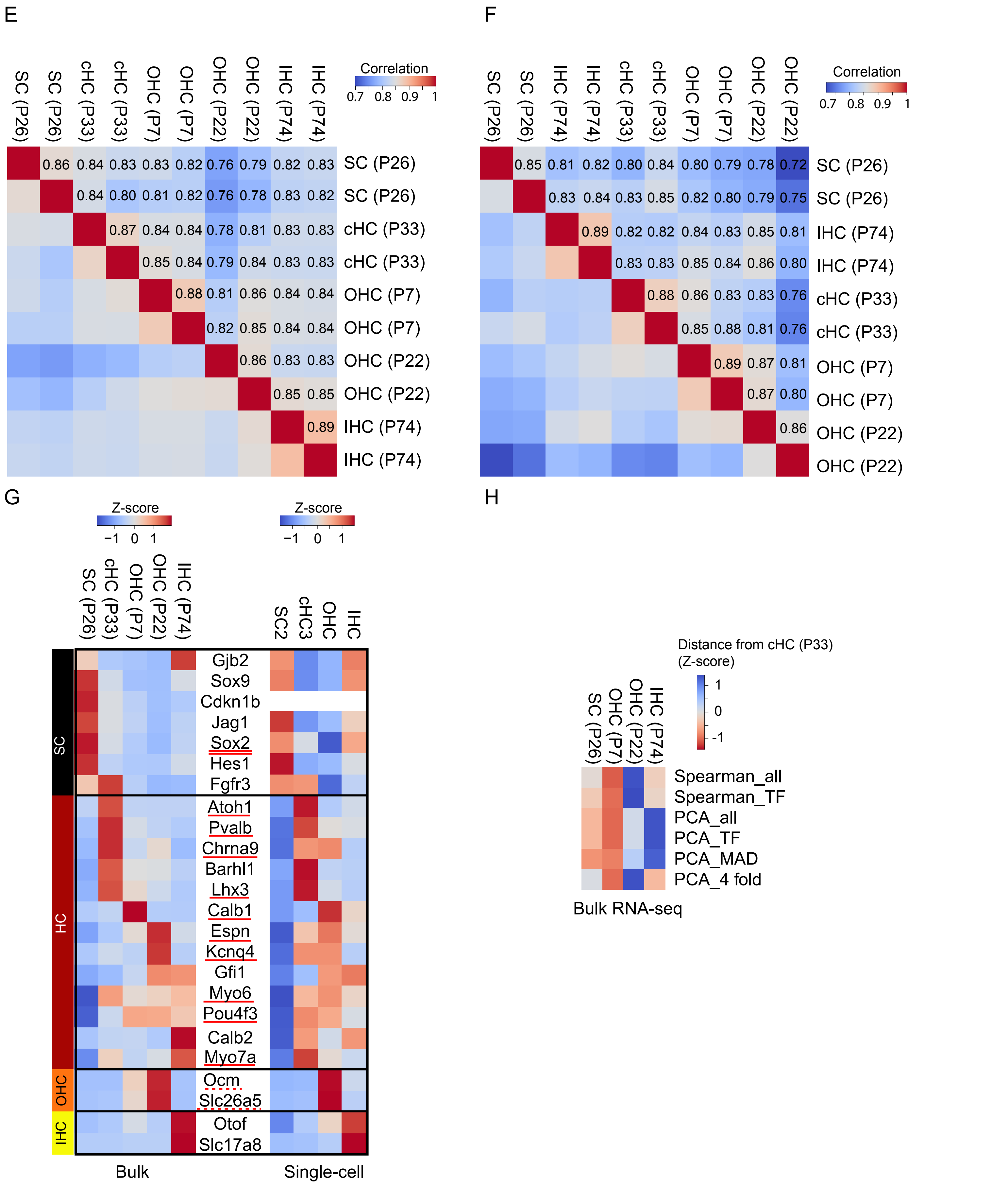

E
F
G
H
Correlation
0.7 0.8 0.9 1
SC (P26)
SC (P26)
cHC (P33)
cHC (P33)
OHC (P7)
OHC (P7)
OHC (P22)
OHC (P22)
IHC (P74)
IHC (P74)
Z-score
−1 0 1
SC2
cHC3
OHC
IHC
SC
HC
Gjb2
Sox9
Cdkn1b
Jag1
Sox2
Hes1
Fgfr3
Atoh1
Pvalb
Chrna9
Barhl1
Lhx3
Calb1
Espn
Kcnq4
Gfi1
Myo6
Pou4f3
Calb2
Myo7a
Ocm
Slc26a5
Otof
Slc17a8
Bulk
Single-cell
Distance from cHC (P33)
(Z-score)
1
0
-1
Spearman_all
Spearman_TF
PCA_all
PCA_TF
PCA_MAD
PCA_4 fold
Bulk RNA-seq

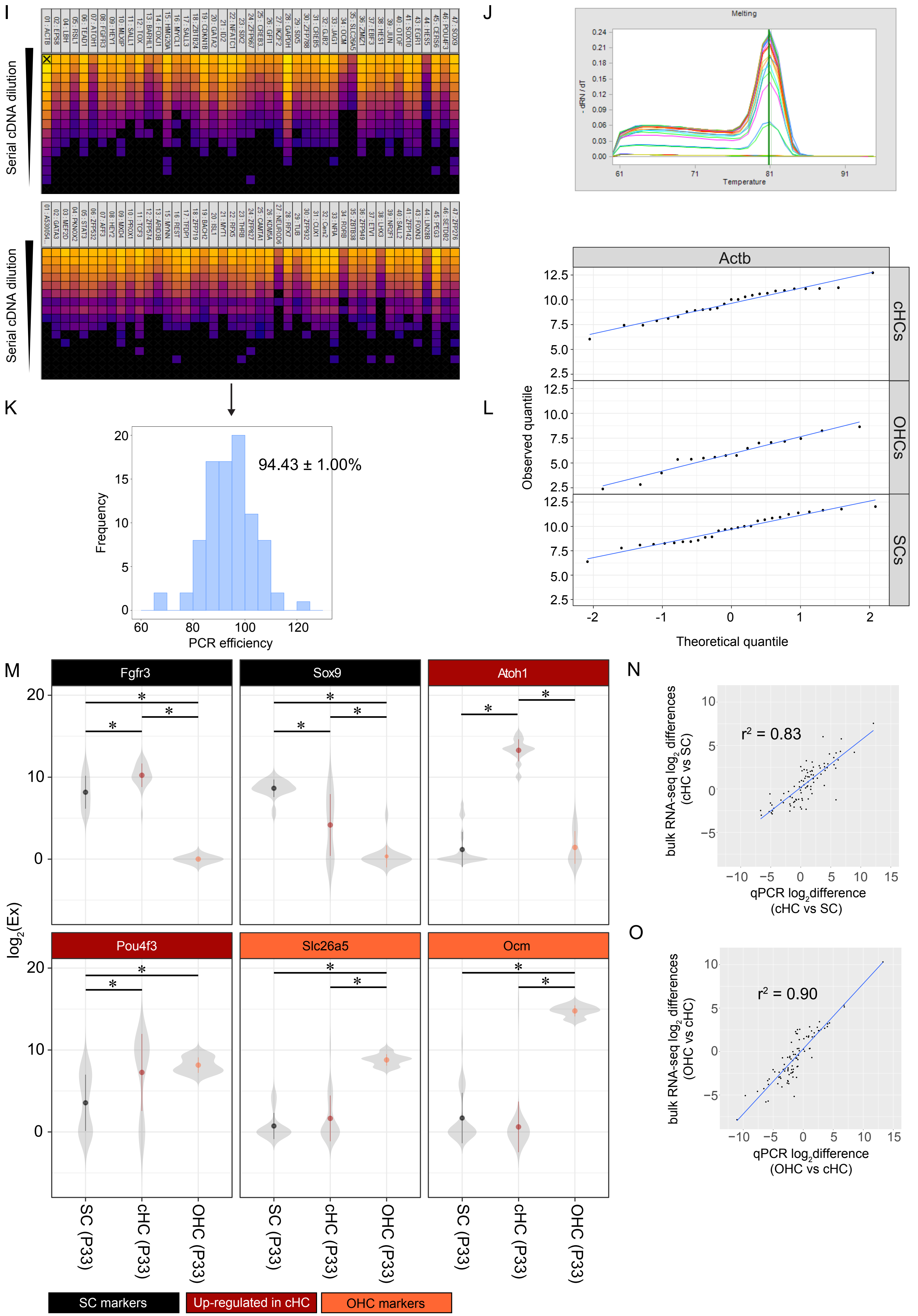

I
Serial cDNA dilution
Serial cDNA dilution
J
Melting
- dRN / dT
Temperature
K
Frequency
PCR efficiency
94.43 ± 1.00%
L
Actb
cHCs
OHCs
SCs
Observed quantile
Theoretical quantile
M
Fgfr3
Sox9
Atoh1
Pou4f3
Slc26a5
Ocm
log2(Ex)
SC (P33)
cHC (P33)
OHC (P33)
SC markers
Up-regulated in cHC
OHC markers
N
r2 = 0.83
bulk RNA-seq log2 differences (cHC vs SC)
qPCR log2difference (cHC vs SC)
O
r2 = 0.90
bulk RNA-seq log2 differences (OHC vs cHC)
qPCR log2difference (OHC vs cHC)

P

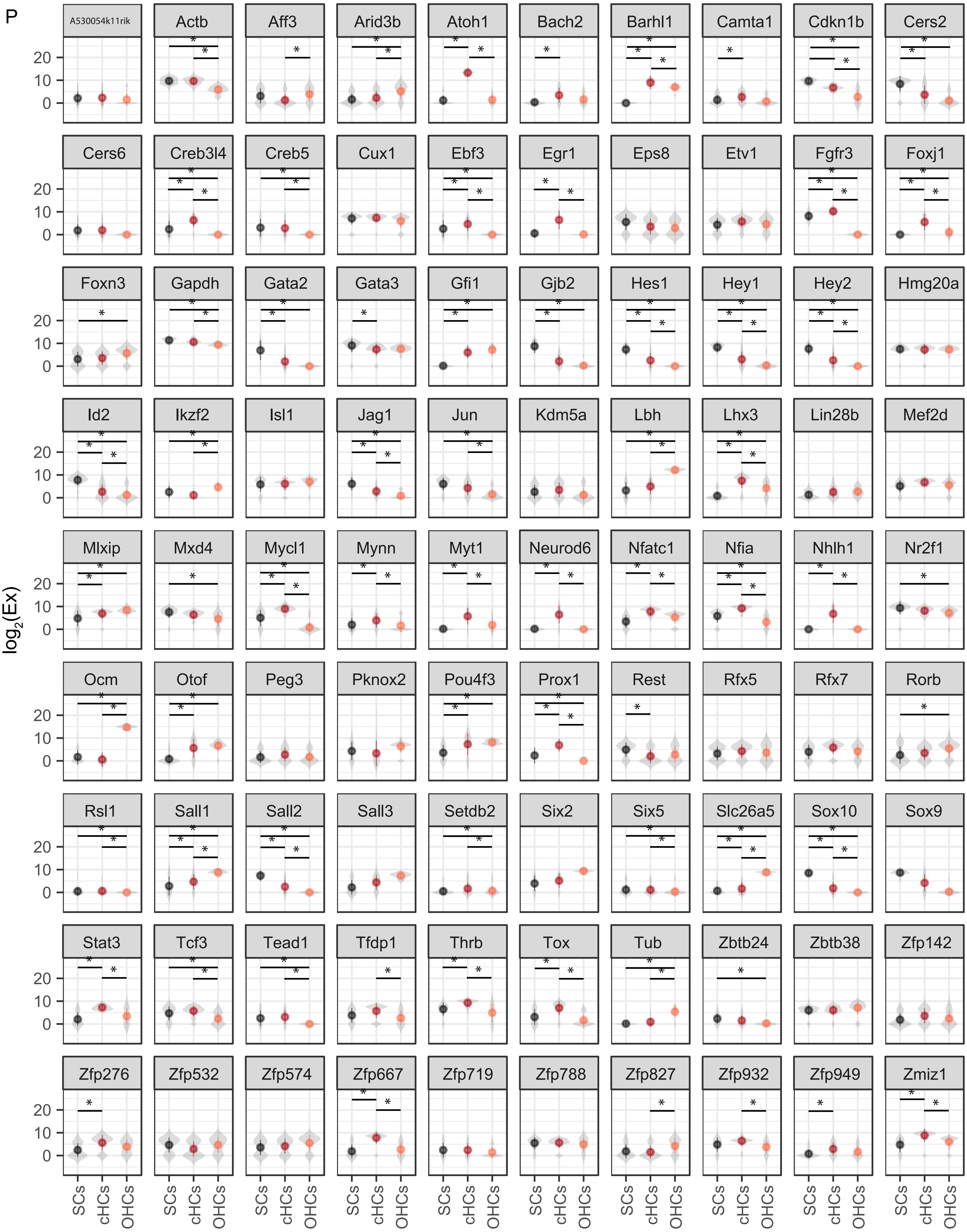
A530054k11rik
Actb
Aff3
Arid3b
Atoh1
Bach2
Barhl1
Camta1
Cdkn1b
Cers2
Cers6
Creb3l4
Creb5
Cux1
Ebf3
Egr1
Eps8
Etv1
Fgfr3
Foxj1
Foxn3
Gapdh
Gata2
Gata3
Gfi1
Gjb2
Hes1
Hey1
Hey2
Hmg20a
Id2
Ikzf2
Isl1
Jag1
Jun
Kdm5a
Lbh
Lhx3
Lin28b
Mef2d
Mlxip
Mxd4
Mycl1
Mynn
Myt1
Neurod6
Nfatc1
Nfia
Nhlh1
Nr2f1
Ocm
Otof
Peg3
Pknox2
Pou4f3
Prox1
Rest
Rfx5
Rfx7
Rorb
Rsl1
Sall1
Sall2
Sall3
Setdb2
Six2
Six5
Slc26a5
Sox10
Sox9
Stat3
Tcf3
Tead1
Tfdp1
Thrb
Tox
Tub
Zbtb24
Zbtb38
Zfp142
Zfp276
Zfp532
Zfp574
Zfp667
Zfp719
Zfp788
Zfp827
Zfp932
Zfp949
Zmiz1
20
10
0
log2(Ex)
SCs
cHCs
OHCs
